# Free-Standing Hierarchically Porous Silica Nanoparticle Superstructures: Bridging the Nano- to Microscale for Tailorable Delivery of Small and Large Therapeutics

**DOI:** 10.1021/acsami.3c16463

**Published:** 2024-01-25

**Authors:** Sandeep Palvai, Delanyo Kpeglo, George Newham, Sally A. Peyman, Stephen D. Evans, Zhan Yuin Ong

**Affiliations:** † School of Physics and Astronomy, University of Leeds, Leeds LS2 9JT, U.K.; ‡ Leeds Institute of Medical Research at St James, School of Medicine, University of Leeds, Leeds LS2 9JT, U.K.

**Keywords:** ice-templating, directional freezing, self-assembly, nanoparticle superstructure, porous silica, controlled drug release, hierarchical porosity

## Abstract

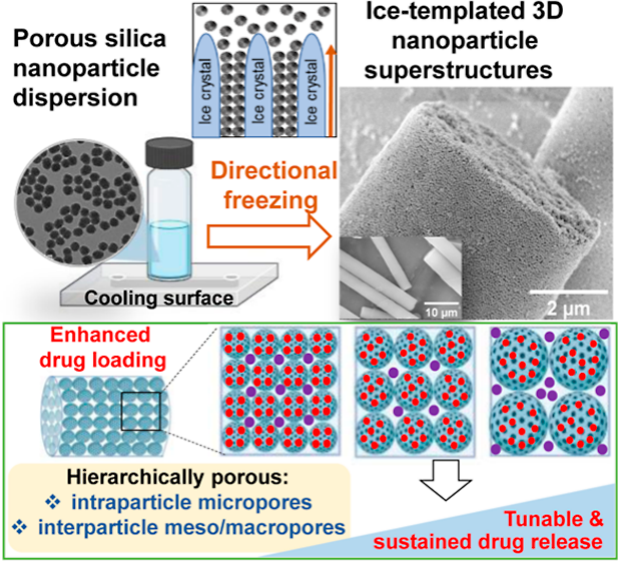

Nanoscale colloidal self-assembly is an exciting approach to yield superstructures with properties distinct from those of individual nanoparticles. However, the bottom-up self-assembly of 3D nanoparticle superstructures typically requires extensive chemical functionalization, harsh conditions, and a long preparation time, which are undesirable for biomedical applications. Here, we report the directional freezing of porous silica nanoparticles (PSiNPs) as a simple and versatile technique to create anisotropic 3D superstructures with hierarchical porosity afforded by microporous PSiNPs and newly generated meso- and macropores between the PSiNPs. By varying the PSiNP building block size, the interparticle pore sizes can be readily tuned. The newly created hierarchical pores greatly augment the loading of a small molecule-anticancer drug, doxorubicin (Dox), and a large macromolecule, lysozyme (Lyz). Importantly, Dox loading into both the micro- and meso/macropores of the nanoparticle assemblies not only gave a pore size-dependent drug release but also significantly extended the drug release to 25 days compared to a much shorter 7 or 11 day drug release from Dox loaded into either the micro- or meso/macropores only. Moreover, a unique temporal drug release profile, with a higher and faster release of Lyz from the larger interparticle macropores than Dox from the smaller PSiNP micropores, was observed. Finally, the formulation of the Dox-loaded superstructures within a composite hydrogel induces prolonged growth inhibition in a 3D spheroid model of pancreatic ductal adenocarcinoma. This study presents a facile modular approach for the rapid assembly of drug-loaded superstructures in fully aqueous environments and demonstrates their potential as highly tailorable and sustained delivery systems for diverse therapeutics.

## 1 Introduction

The hierarchical ordering of biological molecules across multiple length scales in nature has provided inspiration for the fabrication of novel materials with advanced functionalities. One exciting new development in nanotechnology is the creation of 3D nanoparticle superstructures by the bottom-up self-assembly of colloidal nanoparticles via hydrogen bonding, van der Waals forces, hydrophobic interactions, and electrostatic interactions.^1−3^ Such an approach is highly transformative as it promotes the coupling and cooperation between individual organic or inorganic nanoparticles to form more complex, higher-order structures with new collective features that include enhanced optical, mechanical, magnetic, and electronic properties which are absent in discrete nanoparticles or the bulk material. Common methods of preparing 3D nanoparticle superstructures include solvent evaporation, emulsion templating, molecular recognition between nanoparticle surface ligands, the use of biological or synthetic templates, and external magnetic or electric fields.^2,3^ However, these methods typically require a combination of organic solvent usage, extensive chemical functionalization of nanoparticle building blocks (e.g., with oleic acid, dodecylamine, or DNA), long preparation time (days to months), or high temperatures, which could result in the undesirable degradation of bioactive cargoes or induce toxicity due to residual solvents, hence limiting their biomedical applications. Moreover, the need for precise and delicate control over the ligand, solvent, and thermodynamic parameters could also adversely affect the quality, reproducibility, and scalability of the ordered 3D superstructures. There is thus a need for the continued development of less harsh and efficient assembly techniques to broaden the use of hydrophilic nanoparticles with aqueous media and achieve the production of high-quality 3D superstructures for biomedical applications.

**Scheme 1:**
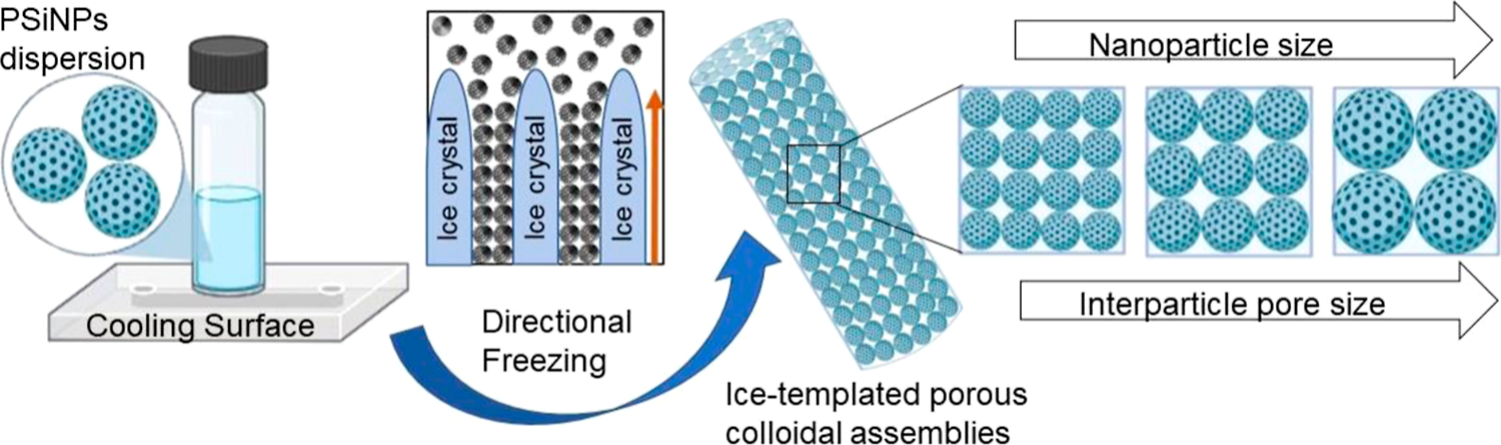
Schematic Diagram Depicting the Directional Freezing Process of PSiNPs and the Formation of 3D Nanoparticle Superstructures The process involves cooling an aqueous dispersion of PSiNPs to −22 °C using a Peltier cooling plate. During this cooling process, ice crystals grow in a unidirectional manner (bottom to top, as depicted), causing the PSiNPs to be compacted in the interstitial spaces between the ice crystals. This compaction leads to the self-assembly of the PSiNPs to form freestanding, organized fibrous 3D nanoparticle assemblies.

In recent years, directional freezing or ice-templating of a dilute colloidal dispersion has emerged as a simple and green method to produce 3D nanoparticle assemblies.^4,5^ During this process, the formation of uniaxially aligned ice crystals serves to expel colloidal nanoparticles from the growing ice front, leading to the organization of the nanoparticles into micron-sized superstructures (Scheme 1).^5−8^ To date, microwires with diameters ranging from 10 to 500 nm have been formed by the directional freezing of a wide range of inorganic (Au, Fe_2_O_3_, ZnO, and InSnO) and organic (polystyrene) nanoparticles.^4,9,10^ However, such unique freestanding 3D superstructures have yet to be exploited for any applications. To the best of our knowledge, we report the first study to utilize directional freezing of discrete porous silica nanoparticles (PSiNPs) to produce anisotropic 3D nanoparticle assemblies with tunable hierarchical porosity conferred by the larger meso/macropores between the PSiNPs and smaller micropores within the PSiNPs for controlling the delivery of small molecules and large macromolecular therapeutics. Microspheres formed by the evaporation-driven assembly of zeolite nanoparticles in water-in-oil emulsions have been previously reported to provide tunable mesopores and hierarchical porosity.^8,11^ Our approach is distinctly different in that unique fibrous 3D nanoparticle assemblies can be achieved within minutes as opposed to days in a fully aqueous environment without the need for heating, calcination, or emulsifiers. As illustrated in Scheme 1, an aqueous dispersion of PSiNPs with approximately 1–2 nm micropores was directionally frozen from the bottom using a Peltier setup. During the freezing process, unidirectionally growing ice crystals drive the compaction and self-assembly of the PSiNPs to collectively create new, larger mesopores between the PSiNPs, thereby imparting distinctive hierarchical porosity to the 3D nanoparticle assemblies. We hypothesized that the hierarchically porous 3D nanoparticle superstructures would permit the loading of one or more types of therapeutics with different molecular sizes into the intraparticle micropores and interparticle meso/macropores, which could in turn afford fine control over the release kinetics of the therapeutics.

Here, we systematically investigated the effect of the nanoparticle building block size, concentration, and freezing temperature (or rate) on the self-assembly of anisotropic 3D nanoparticle superstructures. At optimal freezing rates and sol concentrations, fibrous nanoparticle assemblies with diameters of 3–5 μm were formed. N_2_ gas adsorption–desorption studies revealed hierarchical pore size distribution within the ice-templated 3D nanoparticle superstructures, with small micropores afforded by the PSiNPs and the manifestation of new larger meso/macropores between the PSiNPs. Moreover, the interparticle pore sizes can be readily tuned from 18.5 to 120.9 nm by changing the sizes of the PSiNP building blocks used in the directional freezing process. The newly created hierarchical pores offered an unprecedented opportunity to greatly enhance the loading of an anticancer drug, doxorubicin (Dox), into the micro- and meso/macropores of the 3D nanoparticle superstructures, beyond that achieved with free colloidal PSiNPs. We further demonstrated the ability to coload a larger model therapeutic protein, lysozyme (Lyz), into the interparticle macropores, along with Dox in the smaller intraparticle micropores. Interestingly, a pore size-tunable and sustained drug release was demonstrated with the Dox-loaded superstructures, and a temporal release of Lyz and Dox was seen with the coloaded superstructures. Finally, we developed a hydrogel formulation of Dox-loaded 3D nanoparticle superstructures to aid easy administration at the site of action as an implantable or injectable drug delivery system. A sustained anticancer effect was demonstrated in a 3D cocultured spheroid model of pancreatic ductal adenocarcinoma (PDAC). Overall, this facile modular approach to assembling drug-loaded 3D nanoparticle superstructures by directional freezing effectively bridges the nanoscale to the microscale to create novel materials with highly tunable hierarchical porosity for controlled and temporal drug release applications.

## 2 Experimental Section

### 2.1 Materials

2-Propanol (IPA; 99.7%) was purchased from VWR. l-arginine (Arg), poly(acrylic acid) (PAA; *M*_w_ = 1800), pluronic F-127, and methyl cellulose (Methocel A15 LV; 27.5–31.5% methoxyl basis) were purchased from Sigma-Aldrich. Tetraethoxysilane (TEOS; 99.9%) was purchased from Alfa Aesar. Fluorescein isothiocyanate-poly(ethylene glycol) silane (FITC-PEG-silane; *M*_w_ = 5000) was obtained from Nanocs. Alexa Fluor 350 NHS Ester (Succinimidyl Ester), Calcein, AM, cell-permeant dye, and LIVE/DEAD Fixable Blue Dead Cell Stain Kit for UV excitation were purchased from Thermo Fisher Scientific. Lysozyme (Lyz; hen egg white) was purchased from Merck Life Science Limited. Spectra/Por 7 regenerated cellulose dialysis tubing (1 kDa MWCO) was purchased from Spectrum Laboratories. Doxorubicin HCl (Dox; 95%) was purchased from Fluorochem Limited. All chemicals were of analytical grade and used without further purification. Ultrapure water (Millipore Milli-Q) with an 18.2 MΩ cm resistivity at 25 °C was used in all experiments.

### 2.2 Formation of Porous 3D Nanoparticle Assemblies by Directional Freezing

Porous 3D assemblies of PSiNPs were created by using a controlled, unidirectional freezing technique. Aqueous dispersions of PSiNPs (25, 50, 100, and 150 nm) were prepared at 0.25, 0.5, and 1.0% w/v in 2 mL of ultrapure water. Each sample was sonicated for 1 min to obtain a homogeneous colloidal dispersion. The samples were frozen at −22 °C by using a Peltier thermoelectric cooling module with controlled power adjustments. A thermocouple was used to verify that the surface temperature of the Peltier plate was held at −22 °C during freezing. To achieve freezing at −196 °C, the samples were placed on a surface in contact with liquid nitrogen. Upon freezing, the samples were either lyophilized or thawed to room temperature for further studies. The fibres created from 25, 50, 100, and 150 nm PSiNPs were labeled as Fibre25, Fibre50, Fibre100, and Fibre150, respectively.

### 2.3 Pore Size Distribution Analysis of the Fibres

The pore size distribution of the fibres was determined using N_2_ gas physisorption studies with the Micromeritics Tristar 3000 instrument at a temperature of 77 K. Freeze-dried samples were further dried and degassed under a vacuum at 100 °C for approximately 20 h before porosity analysis. The surface areas and pore volumes were determined using the Brunauer–Emmett–Teller (BET) analysis. The micropore and meso/macro pore size distributions of the fibres were assessed by using the nonlocal density functional theory (NLDFT) and BET analysis methods, respectively. The MicroActive software was used in the data analysis.

### 2.4 Dox Loading into PSiNPs

Dox-loaded PSiNPs were prepared by dispersing 10 mg of PSiNPs in 0.5 mg mL^–1^ of a Dox solution (2.0 mL). The mixture was protected from light and stirred at room temperature for 48 h. The Dox-loaded PSiNPs were separated from the mixture by centrifugation at 17,000*g* for 10 min and then washed three times with ultrapure water to remove any nonencapsulated drugs. To determine the drug loading capacity, the remaining amount of nonencapsulated drug present in the supernatant from each rinse was quantified using UV–vis spectroscopy at 480 nm, with a standard calibration curve made from known Dox concentrations. The total amount of nonencapsulated drug in the supernatant was subtracted from the initial amount of drug added and used to determine the mass of drug that was loaded into the PSiNPs. The drug loading capacity was determined using the following formula
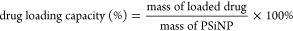


### 2.5 Preparation of Drug-Loaded 3D Assemblies

To load Dox into the interparticle pores of the fibres, a solution of free Dox (0.05% w/v) was mixed with a dispersion of PSiNPs (0.5% w/v) and subjected to directional freezing. The resulting fibres were labeled Inter_Dox_Fibre. Intraparticle Dox-loaded fibres were prepared by freezing the same concentration of Dox-loaded PSiNPs and labeled as Intra_Dox_Fibre. For Dox loading into both the interparticle meso/macropores and intraparticle micropores of the fibres, free Dox (0.05% w/v) was mixed with Dox-loaded PSiNPs (0.5% w/v) and then subjected to directional freezing to give Dual_Dox_Fibre.

Inter_Lyz_Intra_Dox_Fibres100 was prepared by mixing Lyz with Dox-loaded PSiNPs and immediately subjected to directional freezing. All of the drug loading processes were carried out in the dark. After freezing, the samples were thawed, and the fibres were rinsed once with ultrapure water, followed by twice with PBS to remove any nonencapsulated drug. The amount of drug loading was determined using a similar method to that described in the previous section.

### 2.6 Powder XRD Measurement

An X-ray diffractometer (Bruker D2 Phaser operating with Cu Kα radiation, λ = 1.5418 Å, at 300 W) was used to obtain powder XRD (PXRD) patterns for free Dox, nondrug-loaded Fibres100, a physical mixture of Dox and nondrug-loaded Fibres100, Dox@PSiNPs100, Inter_Dox_Fibres100, Intra_Dox_Fibres100, and Dual_Dox_Fibre100. All fibres were analyzed in lyophilized powder form, and the diffraction patterns were recorded over a 2θ range of 5–50°.

### 2.7 Drug Release Studies

To quantify the drug release rate, 5 mg of Dox-loaded fibres was suspended in 1.0 mL of Milli-Q water and transferred to a dialysis bag (1 kDa MWCO). The samples were dialyzed against 30 mL of 1× PBS (pH 7.4) at 37 °C under magnetic stirring. At regular time points, 1.0 mL of the dialysate was removed for analysis of the Dox concentration and replaced with fresh buffer. The amount of drug released was quantified by using UV–vis spectroscopy at 480 nm. To quantify the amount of Lyz and Dox released, 5 mg of Inter_Lyz_Intra_Dox_Fibres were dispersed in 2 mL of PBS, and the amounts of Lyz and Dox released were determined by HPLC analysis.

The Korsmeyer–Peppas model was employed to evaluate the drug release kinetics and mechanisms from the drug-loaded fibres.^12^
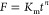
where *F* represents the fraction of drug released, *K*_m_ stands for the kinetic constant, and *n* denotes the release exponent that signifies the drug release mechanism. For this analysis, data points encompassing up to 60% of the drug release within the cumulative release curves were graphed using logarithmic scales. The release exponent *n* was determined from the gradient of the plot, and this *n* value was subsequently introduced into the above equation to determine *K*_m._

### 2.8 Fluorescent Labeling of Lyz

Lyz was reacted with Alexa Fluor 350 NHS ester (2 equiv) in 0.1 M sodium bicarbonate buffer (pH 8.3) in the dark for 2 h at room temperature under constant stirring.^13^ Alexa Fluor 350-labeled Lyz was purified using a Sephadex G-25 column.

### 2.9 Drug Release Time Constant

The drug release time constant for Lyz and Dox was obtained by nonlinear curve fitting; the drug release data was fitted to the following exponential equation
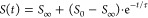
where *S*_∞_ and *S*_0_ stand for the final and initial drug release percentages, *t* indicates time, and τ represents the release time constant.^14^

### 2.10 Composite Hydrogel Formulation

18% w/v PF was dissolved in Mill-Q water at 4 °C and refrigerated overnight to ensure complete dissolution.^15^ Composite hydrogels (cGels) were prepared by mixing 18% w/v PF and 4% w/v MC in Milli-Q water, followed by continued stirring at 4 °C overnight. To formulate drug-loaded fibres in the cGel, 5 mg mL^–1^ of Dual_Dox_Fibre25, Dual_Dox_Fibre100, and Inter_Lyz_Intra_Dox_Fibre100 were added to the PF + MC blend and thoroughly mixed by vortexing before being incubated for 30 min at 37 °C to form the composite hydrogels.

### 2.11 Stability of Hydrogels

The degradation of PF-Gel, cGel, and Fibres100@cGel was evaluated in vitro by using a gravimetric method.^16^ First, the weight of an empty tube was measured and recorded as *W*_v_. Next, 1 mL of PF, PF + MC, or Fibre100 PF + MC blends were added to the tube and incubated at 37 °C for 30 min for gelation. The initial weight of the tube and gel was recorded as *W*_i_. Subsequently, 1 mL of PBS was added to the tube and incubated at 37 °C while being shaken at 200 rpm. At regular intervals, the buffer solutions were removed, and the hydrogels were accurately weighed and recorded as *W*_t_. All experiments were performed in triplicate. The percentage weight of the remaining gel was expressed as
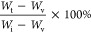


### 2.12 Drug Release from Hydrogels

To assess the drug release rate from the hydrogel formulations, 1 mL of PBS (pH 7.4) was added to Dual_Dox_Fibre25@cGel, Dual_Dox_Fibre100@cGel, and Dox@cGel, and the mixture was left to shake (200 rpm) at 37 °C. At set time points, the PBS was replaced with fresh buffer. The amount of Dox and/or Lyz released was quantified using UV–vis absorption spectroscopy and HPLC.

### 2.13 Swelling Ratio

The components of the Dual_Dox_Fibre25@cGel and Dual_Dox_Fibre100@cGel (0.22 g each) were weighed into individual tubes, and 1 mL of PBS was added to each tube. The formulations were allowed to undergo gelation at 37 °C. At specified time intervals, the swelled samples were carefully weighed after removing the excess liquid to determine their swelling ratio. The same volume of fresh PBS was added to each sample after mass determination. The swelling ratio of the hydrogel formulations was determined using the following equation
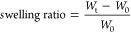
where *W*_t_ represents the final mass of the hydrogels after swelling in PBS, and *W*_0_ denotes the initial mass of the hydrogel samples. All experiments were conducted in triplicate, and the values represent the average ± standard deviation.

### 2.14 Rheology

The rheological properties of PF-Gel, cGel, and Fibres100@cGel were characterized using the Anton Paar MCR 302 rheometer. The hydrogels were freshly prepared and subjected to frequency sweep measurements using an 8 mm parallel plate geometry with a 1.2 mm gap at 20 °C. The temperature was subsequently increased to 37 °C. The measurements were carried out at a 1% shear strain over a 0.1 to 10 Hz frequency range, while strain sweep measurements were performed at a frequency of 1 Hz between 1 and 1000% shear strain. Additionally, the thixotropic properties of the hydrogels were studied using cyclical low (1%) and high (1000%) shear strain periods lasting 30 s at 1 Hz frequency.

### 2.15 Cell Lines and Culture

The human pancreatic ductal adenocarcinoma cell line PANC-1 (ECACC 87092802) and the human pancreatic stellate cells (PSCs)^17^ were chosen for assessment with the Fibre@cGel treatment. PANC-1 cells were cultured in DMEM (Thermo Fisher Scientific) supplemented with 10% FBS (Sigma-Aldrich), 1% penicillin streptomycin (P/S; Sigma-Aldrich), and 1% GlutaMAX (Thermo Fisher Scientific). This will be referred to as the DMEM/10% FBS culture medium. PSCs were cultured with the stellate cell medium supplemented with 10% FBS, 1% stellate cell growth supplement, and 1% P/S in culture flasks coated with poly l-lysine. PSCs and their culturing reagents were sourced from ScienCell Research Laboratories supplied by Caltag Medsystems Ltd. PANC-1 and PSCs were grown at 37 °C with 5% CO_2_ under humidified conditions. They were passaged with TrypLE Express Enzyme with no phenol red (Thermo Fisher Scientific) and used for spheroid culture once ≥70% confluence was achieved.

### 2.16 PDAC Spheroid Culture

Once the PANC-1 and PSCs were ≥70% confluent, the cells were harvested with TrypLE and seeded into Corning 96-well clear round-bottom ultralow attachment (ULA) plates (Scientific Laboratory Supplies) for PDAC spheroid culture at a seeding density of 800 or 1000 cells per well. For the PDAC spheroid culture, PANC-1 and PSC cells were cocultured at a seeding ratio of 1:3. The PDAC spheroids were grown in DMEM/10% FBS culture medium. Bright-field images of the spheroids were taken for size (in width; μm) and volume (mm^3^) assessments using a MATLAB-based SpheroidSizer program to monitor growth.^18^ Once the PDAC spheroids were between 600 and 700 μm in diameter, they were transferred to a 24-well clear flat bottom ULA plate with 1.5 mL of media for assessment with the different Fibre@cGel formulation treatment conditions. Liquid formulations of Inter_Dox_Fibre100@cGel and Dual_Dox_Fibre100@cGel containing 4 μM Dox and a similar quantity of Fibre100@cGel were added to cover the upper surface of the transwell inserts and incubated at 37 °C to form the gels. The gel-containing transwell inserts were then transferred to the 24-well plate containing spheroids. 1 mL of media was removed from the lower compartment at 4 h, days 1 and 3, for the spheroids treated for 7 days and replenished with fresh 1 mL of media. For the spheroids treated for 14 days, the media change was performed at 4 h, days 1, 3 and 7.

### 2.17 WST-1 Cell Viability Assay

The cell viability in spheroids at days 7 and 14 post-treatment was analyzed using the WST-1 assay. The media in each well were replaced with 0.2 mL of growth media and 20 μL of WST-1. The spheroids were incubated for 2 h at 37 °C before the measurement of absorbance at 440 nm using a microplate spectrophotometer (Molecular Devices). Relative cell viability was expressed as [(*A*_sample_ – *A*_blank_)/(*A*_untreated_ – *A*_blank_)] × 100%.

### 2.18 Live/Dead Cell Viability Assessment

After treatment with the different fibre formulations, the PDAC spheroids were incubated with Live/Dead fixable blue dead cell stain for 30 min and Calcein AM for 15 min at 37 °C with 5% CO_2_ according to the manufacturer’s instructions. After incubation, the spheroids were imaged with the Leica-TCS-SP8 confocal laser scanning microscope using a 2.5 or 10× objective and pinhole of 1.00 AU with the respective excitation and emission wavelengths of the stains according to the manufacturer’s instructions. The acquired images were analyzed with ImageJ.

### 2.19 Statistical Analysis

Statistical significance was determined using the two-tailed Student’s *t*-test. The difference between mean values was taken to be statistically significant at *P* < 0.05.

## 3 Results and Discussion

### 3.1 Assembly of PSiNPs into Hierarchically Porous Superstructures by Directional Freezing

Monodisperse PSiNPs of four different sizes were synthesized using our previously reported method for use as building blocks to prepare the 3D nanoparticle superstructures.^19,20^ Detailed information on the synthesis and characterization of the PSiNPs can be found in the Supporting Information. TEM images showed spherical PSiNPs with diameters of 25.9 ± 3.0, 50.9 ± 3.5, 100.5 ± 7.5, and 150.3 ± 8.4 nm (Figure S1A–D). The hydrodynamic diameters of the PSiNPs were found to be between 62.9 and 169.4 nm, with low polydispersity indices (PDIs) of 0.06–0.11 and zeta-potentials between −29 and −33 mV (Figure S1E). For ease of discussion, the diameters of the PSiNPs will be described as 25, 50, 100, and 150 nm in the succeeding paragraphs.

**Figure 1:**
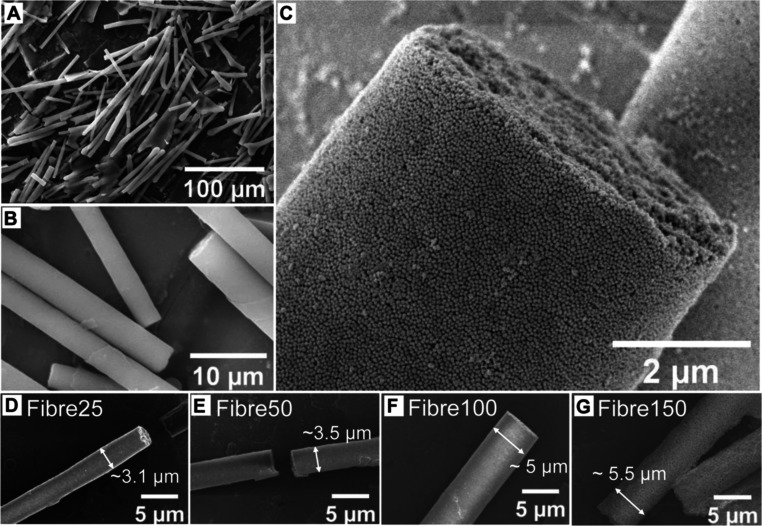
FE-SEM images of 3D nanoparticle superstructures assembled by the unidirectional freezing of 0.5% w/v PSiNPs. (A–C) Fibres assembled from 100 nm PSiNPs seen at low, intermediate, and high magnification, respectively. A progressive increase in fibre diameters was observed when assembled from (D) 25, (E) 50, (F) 100, and (G) 150 nm PSiNPs.

In this study, we investigated the effect of freezing temperature (or rate) and the PSiNP building block concentration on the formation of 3D nanoparticle superstructures by directional freezing. The ice formation during the directional freezing process starts with ice crystal nucleation, followed by growth, with the growth dynamics of the ice crystals playing a crucial role in controlling the morphology of the resulting ice-templated structures. The freezing rate and solute concentration are key variables that affect the ice crystal growth dynamics and morphology of the resulting structure.^21^ PSiNPs were directionally frozen at 0.25, 0.5, and 1.0% w/v in Milli-Q water at −22 and −196 °C (Scheme 1). By freezing the same volume of 0.5% w/v PSiNP dispersion at a higher temperature of −22 °C, a slower rate of freezing (20 μm s^–1^) was observed, whereas a faster rate of freezing (180 μm s^–1^) occurred at the lower temperature of −196 °C. Upon supercooling the PSiNP dispersion, ice nucleation and growth were initiated from the bottom. The PSiNPs were subsequently expelled from the growing ice front and were pushed together in the interstitial spaces between the ice crystals, leading to the self-assembly of 3D nanoparticle superstructures via particle–particle interactions, such as van der Waals attraction and hydrogen bonding (Scheme 1). Slow freezing of 0.5% w/v PSiNP at −22 °C, regardless of the particle sizes, produced free-standing fibrous superstructures after thawing, as confirmed by FE-SEM (Figure 1A,B). As seen at high magnification, PSiNPs were arranged in an orderly manner into fibrous 3D superstructures with newly generated interparticle pores (Figure 1C). In contrast, fast freezing at −196 °C resulted in disorganized lamellar structures (Figure S2). These results are consistent with a previous study by Yan et al., who observed lamellar structures at a faster freezing rate and fibres at a slower freezing rate when an appropriate concentration of polystyrene nanoparticle dispersion was directionally frozen.^22^ In our study, the total freezing time for 2 mL of PSiNPs dispersion was approximately 14–17 and 1–2 min at −22 and −196 °C, respectively (Table S1). The essential process for forming organized ice-templated assemblies involves the rejection of particles from the growing ice crystals. Typically, a higher freezing rate induces swift ice nucleation, resulting in the formation of abundant, significantly smaller ice crystals lacking a specific growth orientation.^23^ In contrast, slower freezing permits the ice nuclei to develop into larger, aligned ice crystals, facilitating the concentration and compaction of sol particles within the interstitial spaces to form large fibrous nanoparticle assemblies. A relationship between the solidification velocities (*v*) of the growing ice crystals and critical freezing front velocity (*v*_cr_) has been previously employed to predict whether the particles will be rejected or entrapped by the growing ice front.^24,25^ Particles are generally rejected by the ice front when *v* < *v*_cr_, while they are entrapped within the growing ice crystals due to insufficient time for their segregation from the suspension when *v* ≫ *v*_cr_. Similarly, our study demonstrated that a slower freezing of colloidal dispersions at an optimum colloidal concentration allows sufficient time for the PSiNPs to self-organize within the interstitial boundaries of growing ice crystals, leading to the formation of well-organized fibrous 3D nanoparticle assemblies. Conversely, rapid freezing at −196 °C could have led to PSiNP entrapment within the ice crystals and reduced the organization and compaction of PSiNPs within the interstitial spaces of the ice fingers, resulting in the formation of random aggregates.^22,25,26^

Additionally, directional freezing of 0.25 and 1.0% w/v PSiNPs did not yield uniform fibres but instead gave a mix of fibres with other structures such as spheres, short rods, and ellipsoids (Figure S3). Therefore, we fixed the PSiNP concentration at 0.5% w/v and directionally froze the samples at −22 °C to form the 3D nanoparticle superstructures for all subsequent studies. Fibres assembled from 25, 50, 100, and 150 nm PSiNPs were denoted as Fibres25, Fibres50, Fibres100, and Fibres150, respectively. The resulting fibres had diameters ranging from 3.1 to 5.5 μm (Table 1 and Figure 1D–G). Increasing the diameter of the PSiNPs used in the fibre assembly process from 25 to 150 nm resulted in larger fibre diameters. However, regardless of fibre size, we did not see a significant correlation between the PSiNP diameter and fibre lengths in the assembled structures. Instead, a wide length distribution ranging from 40 to 150 μm was observed in all the fibres. The stability of the fibres was investigated by incubation in PBS and evaluating changes in morphology using FE-SEM over time. It was found that the fibres remained structurally intact and did not undergo significant disassembly, with average diameters remaining largely unchanged for up to 45 days (Figure S4).

**Table 1:** Diameters of Fibres Determined by FE-SEM (*n* = 200) and Porosity Data Obtained by N_2_ Gas Adsorption–Desorption Studies with BJH and BET Analyses

fibre type	diameter [μm]	pore volume [cm^3^ g^–1^]	surface area [m^2^ g^–1^]
Fibre25	3.1 ± 0.4	0.41	143.3
Fibre50	3.5 ± 0.5	0.41	127.5
Fibre100	5.0 ± 0.6	0.34	44.3
Fibre150	5.5 ± 0.8	0.34	37.9

### 3.2 Porosity Analysis of Fibres

**Figure 2:**
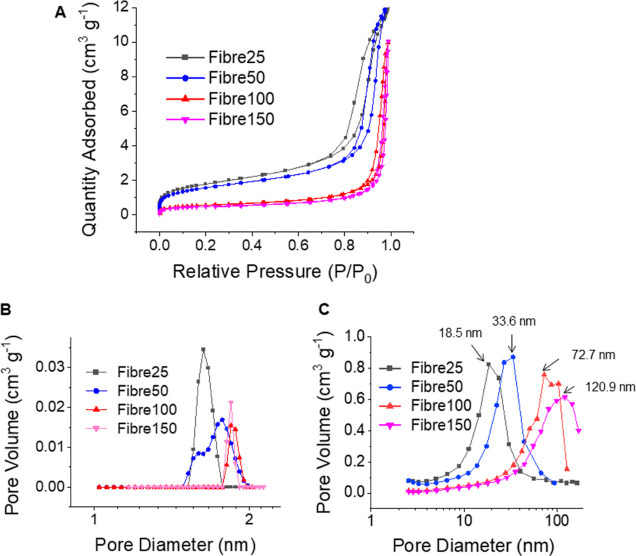
(A) N_2_ gas adsorption/desorption isotherms collected at 77 K for the 3D nanoparticle assemblies and the corresponding pore size distributions determined using (B) nonlocal density function theory analysis for the micropores and (C) BJH analysis for the meso/macropores.

To evaluate the porosity of the as-formed fibres, N_2_ gas absorption–desorption studies were performed. The IUPAC-type IVa isotherms observed in Figure 2A are indicative of adsorption behavior within mesoporous adsorbents. As evident from all the isotherms, the rapid N_2_ gas uptake at *P*/*P*_o_ < 0.1 indicates the presence of micropores. Additionally, the isotherms display a hysteresis loop region at high *P*/*P*_o_, which signifies the existence of mesopores. Moreover, the observed type H3 hysteresis loops without a limit of adsorption at high *P*/*P*_o_ suggest the presence of pores corresponding to the aggregates of the particles, clearly demonstrating the generation of new interparticle meso/macro pores during the self-assembly of PSiNPs.^27^ The parallel alignment of the adsorption and desorption branches of the hysteresis loop is indicative of an open mesoporous structure extending to the outer surface.^28,29^ The hierarchical porosity of the fibres was confirmed using a combination of NLDFT and BJH analyses. First, NLDFT analyses revealed the presence of 1.6–1.9 nm micropores within the fibres (Figure 2B), which closely correspond with the 1.3–2.6 nm intraparticle micropores of the PSiNPs (Figure S5). BJH analyses were performed to analyze the interparticle meso/macropores generated through the self-assembly of the PSiNPs. As seen from Figure 2C, an increase in the diameter of the PSiNP building blocks from 25 to 150 nm resulted in a progressive increase in the average peak diameters of the interparticle pores from 18.5 to 120.9 nm. These results were consistent with the findings of Fijneman et al., for which the dense packing of larger 25 nm silica nanoparticles into microspheres via evaporation-driven self-assembly created larger interparticle void spaces and average pore diameters of ∼40 nm compared to the ∼5 nm pores formed by the smaller 4 nm nanoparticles.^8^ Furthermore, the hysteresis loops became steeper as the PSiNP size was increased in the Fibre25 to Fibre150 samples, indicating a faster rate of nitrogen gas adsorption/desorption from the larger interparticle pores found in fibres assembled from the larger PSiNPs.^8^ In this study, the pore volumes of fibres assembled from smaller PSiNPs (Fibre25 and Fibre50) exhibited higher pore volumes compared to those assembled from larger PSiNPs (Fibre100 and Fibre150) (Table 1). The surface area of the fibres was also found to increase when the PSiNP building block size was reduced. In contrast, the average diameter of the fibres was observed to increase with the size of the PSiNP building block used in the self-assembly process (Table 1). The ability to vary the average interparticle pore diameters from the mesoporous (2–50 nm) to the macroporous (>50 nm) region within the 3D nanoparticle assemblies while retaining the intraparticle microporosity by simply varying the size of the monodisperse PSiNP building blocks offers opportunities for tailoring the loading and release of different macromolecular therapeutics without requiring post-treatment steps.

### 3.3 Drug Loading and Tunability of Drug Release with Change in Porosity

**Figure 3:**
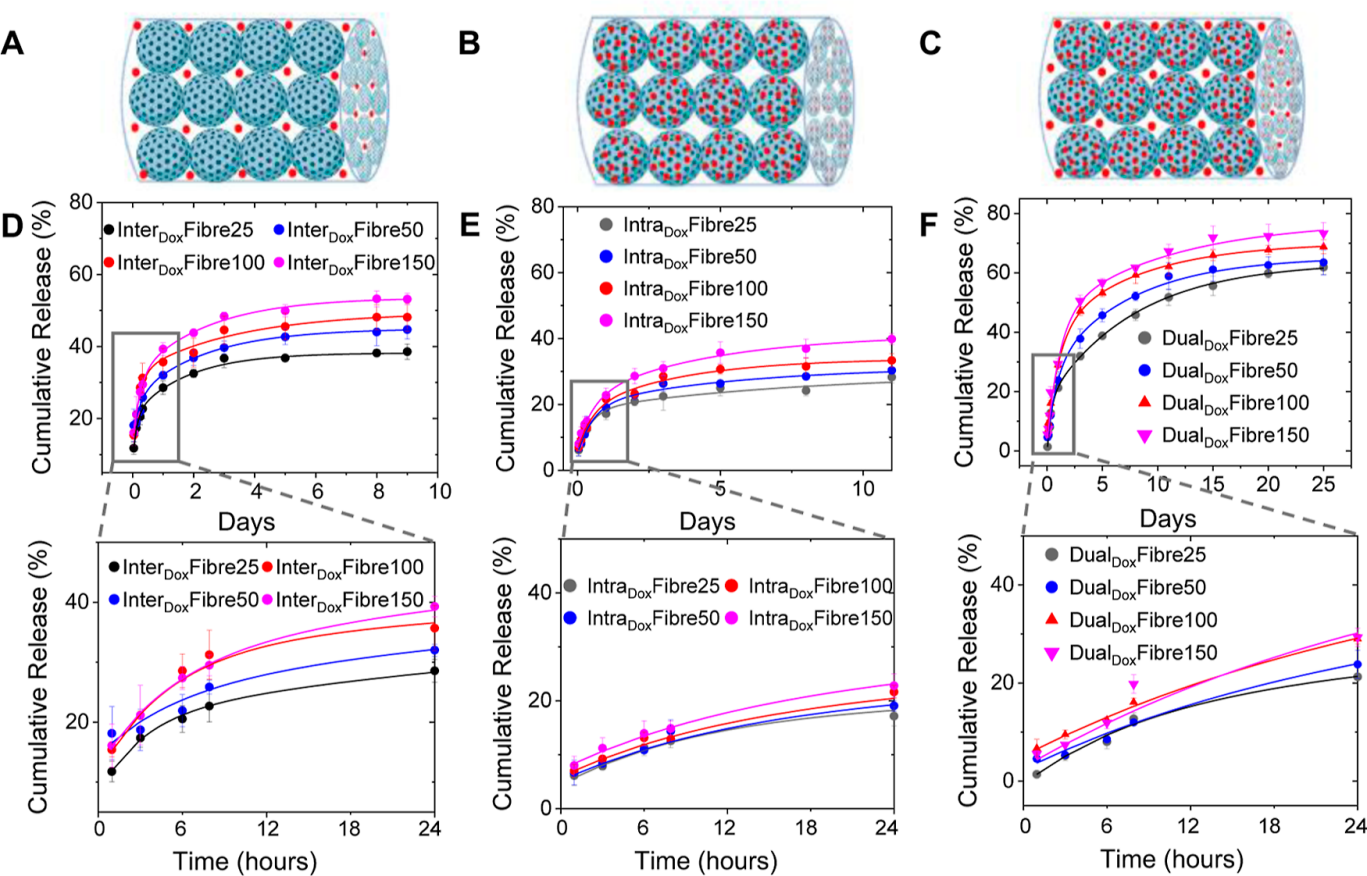
Fibres with Dox loaded in (A) interparticle meso/macropores (Inter_Dox_Fibres), (B) intraparticle pores of the PSiNPs (Intra_Dox_Fibres), and (C) both inter- and intraparticle pores (Dual_Dox_Fibres). Cumulative Dox release from (D) Inter_Dox_Fibres, (E) Intra_Dox_Fibres, and (F) Dual_Dox_Fibres in pH 7.4 PBS at 37 °C. The results represent the mean ± standard deviation of three replicates.

In this study, we propose that the hierarchically porous fibres, with their combination of smaller nanoparticle micropores and larger interparticle meso/macropores, can regulate drug release rates effectively. This feature holds promise for precise control over release kinetics, tailored to specific disease conditions. To test this hypothesis, the small-molecule anticancer drug doxorubicin (Dox) was used as a model drug. Dox was loaded into the hierarchically porous fibres in three different ways: (1) larger interparticle meso/macropores (Inter_Dox_Fibre) by simply mixing PSiNPs with free Dox solution and immediately subjecting the mixture to directional freezing (Figure 3A); (2) smaller intraparticle micropores of the PSiNPs (Intra_Dox_Fibre) by preloading Dox into the PSiNPs micropores before directionally freezing the Dox-loaded PSiNPs (Figure 3B); and (3) combination of larger interparticle spacing and smaller PSiNP micropores of the fibres (Dual_Dox_Fibre) by directionally freezing a mixture of free Dox with Dox-loaded PSiNPs (Figure 3C). Under FE-SEM, the diameters and fibrous morphology of the nanoparticle superstructures remained largely unchanged after drug loading (Figure S6). The directional freezing method yielded a high and near quantitative drug encapsulation efficiency of ≥96.4% in the Inter_Dox_Fibres assembled from PSiNPs with different diameters due to efficient Dox entrapment in the interparticle spaces (Table S2). Consistent with the increase in interparticle pore volumes (Table 1), an appreciable stepwise increase in Dox loading was observed when larger PSiNPs were used in the self-assembly of Inter_Dox_Fibres and Dual_Dox_Fibres (Table 2). These results suggest a greater packing and incorporation of Dox in the larger interparticle void spaces of the fibres as the size of the PSiNPs used in the self-assembly process increased. A smaller increase in Dox loading into the intraparticle pores was also seen when larger PSiNPs were used to assemble the Intra_Dox_Fibres. This could be explained by an increase in drug loading into the intraparticle pores of the PSiNPs as their diameters increased (Table S3). Crucially, Dual_Dox_Fibres gave ∼1.5-fold and 3-fold higher Dox loading content compared to Intra_Dox_Fibres and Inter_Dox_Fibres, respectively, when comparably sized PSiNPs were used in the self-assembly. This result clearly shows that the directional freezing of PSiNPs to form hierarchically porous 3D superstructures offers the added benefit of enhancing drug loading beyond that achieved with individual nanoparticles. The successful encapsulation of Dox into the fibres was confirmed by PXRD. As seen from Figure S7, sharp crystalline peaks between 16 and 27° were observed for nonencapsulated Dox as well as a physical mixture of Dox with Fibre100 (no drug encapsulated).^30^ In comparison, the PXRD spectra of Dox-loaded PSiNPs (Dox@PSiNP100), Inter_Dox_Fibre100, and Dual_Dox_Fibre100 did not show characteristic Dox crystalline peaks and were similar to those of the blank Fibre100. These results strongly suggest that Dox was encapsulated within the intraparticle and/or interparticle pores of the fibres as opposed to being physically adsorbed on the surface.

**Table 2:** Drug Loading Content (μg mg^–1^) of Fibres Formed from PSiNPs of Different Sizes and with Dox Loaded in the Interparticle Mesopores (Inter_Dox_Fibre), PSiNPs Intraparticle Micropores (Intra_Dox_Fibre), and in Both the Interparticle Meso/Macropores and Intraparticle Micropores of the PSiNPs (Dual_Dox_Fibre)

PSiNP diameter (nm)	Inter_Dox_Fibre	Intra_Dox_Fibre	Dual_Dox_Fibre
25	42.7 ± 0.3	93.4 ± 1.2	140.3 ± 2.8
50	47.8 ± 1.1	96.8 ± 2.8	147.5 ± 1.6
100	51.9 ± 1.0	99.6 ± 0.1	153.8 ± 1.8
150	58.4 ± 0.4	103.3 ± 1.5	158.0 ± 2.8

A detailed exposition of the effect of hierarchical pores within the nanoparticle assemblies on drug release was investigated in 1× PBS (pH 7.4). First, drug release from Inter_Dox_Fibres, in which Dox was loaded into the interparticle spaces between the PSiNPs, correlated with an increase in the size of the PSiNP building blocks (Figure 3D) and therefore the size of the interparticle mesopores, as discussed in Section 2.2. Following an initial burst release, the amount of Dox released gradually increased to 38.2, 44.0, 48.2, and 53.3% for Inter_Dox_Fibre25, Inter_Dox_Fibre50, Inter_Dox_Fibre100, and Inter_Dox_Fibre150, respectively, by day 8, with minimal drug released thereafter. These results suggest that the larger interparticle meso/macropores provided a greater ease of Dox dissolution due to increased water penetration and reduced steric hindrance within the pores, leading to the enhanced diffusion of Dox from the nanoparticle assemblies to the external environment. Second, Intra_Dox_Fibres, in which Dox was loaded only into the intraparticle micropores of the PSiNPs, showed a much slower initial drug release than the Inter_Dox_Fibres, with Dox release eventually plateauing at lower levels of 28.3, 30.4, 33.4, and 39.9% for Intra_Dox_Fibre25, Intra_Dox_Fibre50, Intra_Dox_Fibre100, and Intra_Dox_Fibre150, respectively, by day 11 (Figure 3E). The slower release profiles seen with the Intra_Dox_Fibres could be attributed to greater Dox confinement within the disordered PSiNP micropores, leading to a more restricted initial diffusion of Dox into the meso/macroporous void spaces between the PSiNPs. The interparticle pores subsequently provided a secondary level of drug diffusion control, with a faster and greater amount of Dox released as the size of the PSiNP building block, and therefore, the mesopore volumes were increased. More importantly, Dual_Dox_Fibres, in which Dox was loaded in both the interparticle mesopores, and PSiNP micropores showed a slower drug release compared to the Inter_Dox_Fibres and Intra_Dox_Fibres for the first 6–8 h, beyond which a significantly prolonged release of Dox for up to 25 days was observed (Figure 3F). The total amount of Dox released also reached much higher levels at 61.9, 63.5, 68.8, and 73.3% for Dual_Dox_Fibre25, Dual_Dox_Fibre50, Dual_Dox_Fibre100, and Dual_Dox_Fibre150, respectively. These results clearly demonstrate the ability to tune the drug release kinetics and confer a more desirable slow, extended drug release profile via selective drug loading into the micropores and meso/macropores, as well as variation of the PSiNP building block size within the hierarchically porous nanoparticle assemblies.

The Dox release kinetics and mechanism were further investigated by fitting the release data to the Korsmeyer–Peppas model.^12,31,32^ In all cases, *R*^2^ values of ≥0.93 were obtained, indicating a good fit between the experimental data and the model (Table 3). In general, an increase in the diameter of the PSiNP building blocks used in the assembly of each Dox-loaded formulation led to an increase in the kinetic constant, *K*_m_. This result indicates faster drug release rates from the fibrous nanoparticle superstructures with larger interparticle meso/macropores. The calculated release exponent values (*n*) for Inter_Dox_Fibres and Intra_Dox_Fibres were found to be less than 0.45, indicating a Fickian transport mechanism where drug release from these fibres is primarily controlled by diffusion.^33^ Higher *K*_m_ values were observed with the Inter_Dox_Fibres compared to the Intra_Dox_Fibres. The former contains Dox loaded in the meso/macropores, while Dox was loaded into the microporous PSiNPs of the latter. This finding is thus consistent with higher diffusivity rates observed in mesopores than micropores in other studies.^34^ Interestingly, our study showed that the n values of the Intra_Dox_Fibres were higher than those of Inter_Dox_Fibres formed from the same PSiNP diameter. In particular, the n values of the Intra_Dox_Fibres formed by the smaller 25 and 50 nm PSiNPs were increased to 0.44 and 0.42, respectively, which are close to the boundary between Fickian and non-Fickian diffusion when compared to those assembled from larger PSiNPs. Dox molecules have a maximum diameter of 1.5 nm, which is just slightly smaller than most of the ∼1.3–2.6 nm micropores of the PSiNPs used to form the Intra_Dox_Fibres (Figure S5B).^35^ They are thus expected to show single-file diffusion dynamics and have a more restricted passage due to enhanced collisions with the walls of the micropores, leading to slower drug release rates.^34,36^ In the hierarchically porous Intra_Dox_Fibres, the Dox molecules then have to diffuse from the microporous PSiNPs into the interparticle mesopores, where they may encounter greater molecular interactions with each other and the walls of the pores as the void spaces are reduced with the use of smaller PSiNP building blocks. This plausibly led to a reduction in *K*_m_ values and increased *n* values, albeit still maintaining Fickian transport mechanisms. In contrast, the presence of Dox in both the PSiNP micropores and the interparticle meso/macropores of the Dual_Dox_Fibres gave rise to an anomalous or non-Fickian mechanism of drug release, with *n* values >0.45. In the Dual_Dox_Fibres, a higher proportion of Dox (approximately 70% of total Dox) was loaded into the PSiNP micropores compared to the interparticle meso/macropores. Consequently, the diffusion of Dox from the micropores was likely hindered by the presence of Dox in the interparticle meso/macropores. This scenario is in contrast to that encountered with the Intra_Dox_Fibres where rapid Fickian diffusion of Dox from the micropores into much larger and initially unoccupied meso/macropores could occur. Interestingly, the drug release mechanism observed with the Dual_Dox_Fibres in our study appears to be consistent with the non-Fickian transport of various small organic solvent molecules through interconnected micro- and mesopores of hierarchically porous zeolites,^34,36^ hence suggesting interconnectivity of the pores within the 3D nanoparticle assemblies.

**Table 3:** Evaluation of Drug Release Kinetics Using the Korsmeyer–Peppas Model

fibre type	*n*	*K*_m_	*R*^2^
Inter_Dox_Fibre25	0.22	26.77 ± 1.10	0.96
Inter_Dox_Fibre50	0.20	31.70 ± 0.60	0.97
Inter_Dox_Fibre100	0.22	33.50 ± 0.92	0.97
Inter_Dox_Fibre150	0.22	38.20 ± 0.70	0.95
Intra_Dox_Fibre25	0.44	18.92 ± 0.92	0.93
Intra_Dox_Fibre50	0.42	19.37 ± 0.91	0.98
Intra_Dox_Fibre100	0.33	19.31 ± 0.36	0.94
Intra_Dox_Fibre150	0.34	22.41 ± 0.959	0.99
Dual_Dox_Fibre25	0.65	20.80 ± 0.30	0.99
Dual_Dox_Fibre50	0.53	22.80 ± 0.60	0.98
Dual_Dox_Fibre100	0.57	28.17 ± 0.97	0.97
Dual_Dox_Fibre150	0.67	32.11 ± 1.15	0.98

**Figure 4:**
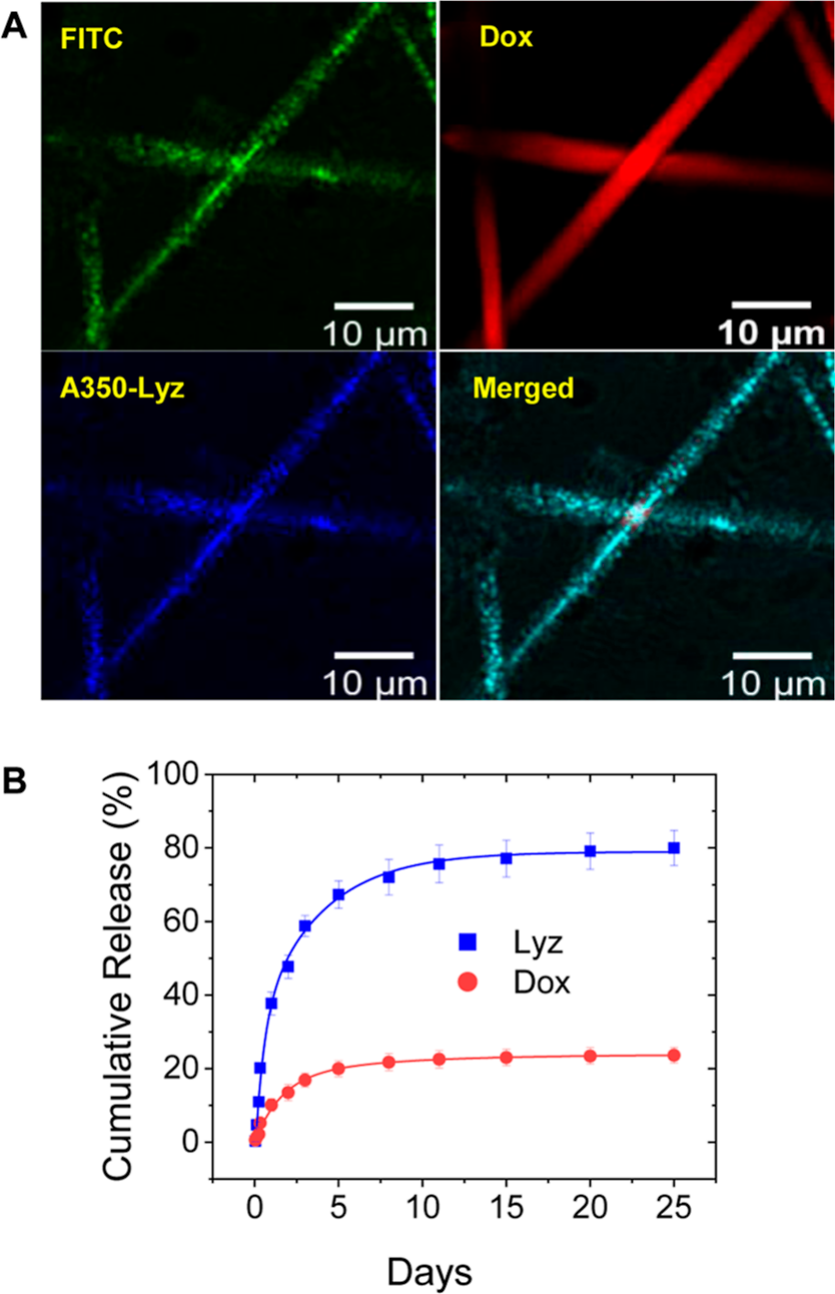
(A) Confocal fluorescence images showing fibres assembled from FITC-labeled PSiNPs (100 nm), which were coloaded with the small molecule drug, doxorubicin (Dox), within the microporous PSiNPs and a large macromolecular protein, Alexa Fluor 350-labeled lysozyme (A350-Lyz), within the interparticle macropores. The merged image shows the colocalization of PSiNPs with both molecules within the fibrous superstructures. (B) Cumulative release of Lyz and Dox over time in pH 7.4 PBS at 37 °C (*n* = 3).

To further demonstrate the versatility of this technique for the loading of larger therapeutic molecules, the model protein, lysozyme (Lyz, 14.7 kDa), was directionally frozen with Dox-loaded PSiNPs (100 nm) to achieve coloading of Lyz in the larger interparticle macropores and small molecule Dox in the PSiNP micropores. Lyz has reported dimensions of 4.5 × 3.5 × 3.5 nm, which are significantly smaller than the 72.7 nm average interparticle pore diameter found in Fibre100 (Figure 2C).^37^ As seen from Figure S8A, the fibrous morphology of the nanoparticle assemblies was retained after coloading of Lyz and Dox without a significant change in fibre diameters. To show colocalization of the two differently sized therapeutics within the nanoparticle assemblies, we prepared FITC-labeled PSiNPs (FITC-PSiNPs) and labeled Lyz with the Alexa Fluor 350 dye (A350-Lyz). Confocal fluorescence images of fibrous superstructures assembled from Dox-loaded FITC-PSiNPs and with A350-Lyz in the interparticle macropores showed the colocalization of green (FITC-PSiNP), red (Dox), and blue (A350-Lyz) signals, which clearly indicate the successful coloading of Lyz and Dox (Figure 4A). The loading contents of Lyz and Dox were found to be 75.7 ± 9 and 114.4 ± 2 μg mg^–1^, respectively (Figure S8B). Subsequent cumulative release studies in PBS showed a more rapid Lyz release from the interparticle macropores, with cumulative releases of 37.7, 72.0, and 80.0% observed on days 1, 8, and 25, respectively, while Dox displayed a much slower release profile, with cumulative releases of 10.0, 21.7, and 23.6% on the same days (Figure 4B). The time constants (τ) for Lyz and Dox releases were found to be 20.8 and 41.0 h, respectively. The higher time constant values observed for Dox release further confirmed the delayed drug release from the smaller intraparticle micropores. In contrast, Lyz release demonstrated a lower time constant, which indicated a faster drug release from larger interparticle macropores. These results thus demonstrate that the hierarchically porous 3D nanoparticle superstructures have the potential for the combined and temporal delivery of therapeutics, which could improve the safety and efficacy of cancer treatments. One such example could be the sequential delivery of a large-molecule protein (e.g., TRAIL or the p53 tumor suppressor gene) or gene to sensitize cells to the subsequent release of a small-molecule anticancer drug.^38^

### 3.4 Fibre-Loaded Composite Hydrogel Formulation

While the self-assembled fibrous nanoparticle superstructures provide precise control over drug release, an adaptable matrix that facilitates the ease of application and promotes adherence to tissues in the body is necessary to enhance the therapeutic outcome. For instance, Gliadel wafer, which is the only FDA-approved implant for postsurgical use to reduce glioma recurrence, possesses limitations due to the inherent rigidity of the polymeric wafer.^39^ This impedes the adherence of the wafers to the tissue walls of the resection cavity, resulting in uneven and unpredictable drug release. Conversely, hydrogel-based drug delivery systems possess desirable viscoelastic properties that allow conformity and adherence to the irregular tumor resection cavity walls. As such, the formulation of the fibres within a biocompatible hydrogel formulation could potentially facilitate the successful implantation of the localized drug delivery system at the intended site of action to enhance the safety and efficacy of future in vivo applications.

**Figure 5:**
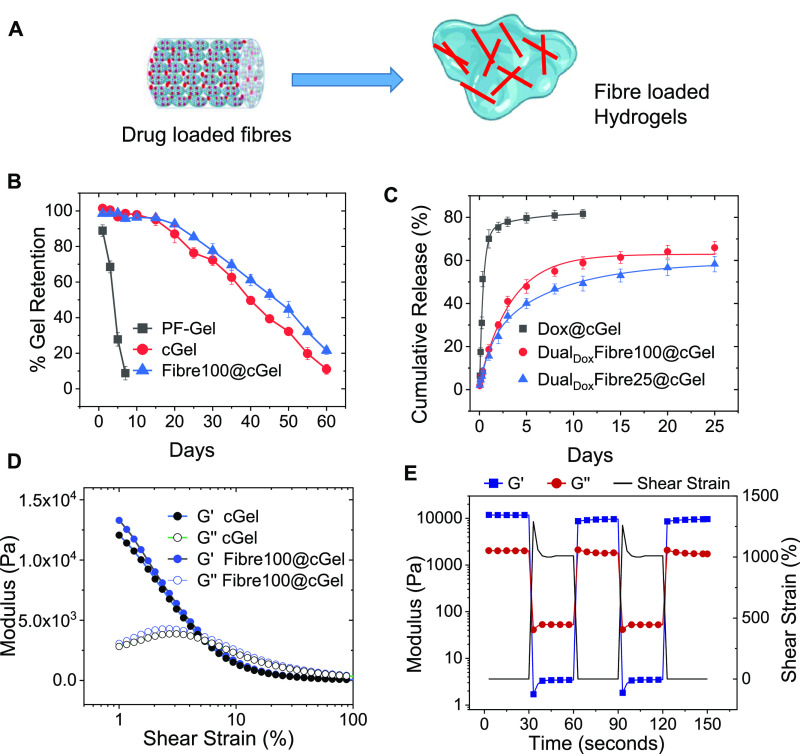
(A) Schematic representation of Dual_Dox_Fibre incorporation into the Pluronic F127 (PF) and methylcellulose composite hydrogel formulation (cGel). (B) Stability of hydrogel formulations in PBS at 37 °C. (C) Release profiles of Dox from cGel formulations in pH 7.4 PBS at 37 °C (*n* = 3). (D) Strain sweep analysis of cGel and Fibre100@cGel at a frequency of 1 Hz. (E) Storage modulus of Fibre100@cGel (measured at a frequency of 1 Hz) under repeated cycles of 1 and 1000% strain with an interval time of 30 s.

We thus prepared a composite hydrogel (cGel) by mixing the fibres with Pluronic F127 (PF), a triblock copolymer composed of poly(ethylene oxide)–poly(propylene oxide)–poly(ethylene oxide), and methylcellulose (MC) (Figure 5A). PF exhibits thermoreversibility based on micelle aggregation, transitioning from a sol state at room temperature to a gel state at physiological temperature. However, PF-only hydrogels (PF-Gel) are unstable due to their fast dissolution in aqueous solutions.^15,40^ To increase stability, 4% w/v MC was added to 18% w/v PF to make cGel. The cGel showed >75% gel retention for up to 25 days, while most of the PF-Gel dissolved within a week in PBS (Figure 5B). The incorporation of the fibrous nanoparticle superstructures in the cGel (Fibre100@cGel) slightly enhanced gel retention for up to 60 days, demonstrating that the fibres did not adversely disrupt the polymer networks and interfere with the stability of the hydrogel.

Dual_Dox_Fibres@cGels showed a very slight reduction in the quantity of Dox released over time compared to the Dual_Dox_Fibre (i.e., 65.9 vs 68.8% at day 25; Figure 5C), which could be due to hydrogen bonding and hydrophobic interactions of the released Dox with the PF and MC within the cGels. More importantly, the trend in which a faster and higher amount of Dox was released from the Dual_Dox_Fibre100@cGel compared to the Dual_Dox_Fibre25@cGel consistent with that seen in fibres with larger interparticle mesopores in Section 2.3, was maintained (Figure 5C). This result indicates that the pore-size-dependent drug release control of the fibres was preserved in the hydrogel formulation. The release profile demonstrated an initial rapid release of up to 15.5 and 18.6% within 24 h, followed by slow release of up to 58.2 and 65.9% until 25 days from Dual_Dox_Fibre25@cGel and Dual_Dox_Fibres100@cGel, respectively. In contrast, a large burst release of Dox (80% at day 5) was observed from the free Dox-loaded hydrogel (Dox@cGel; Figure 5C). The effect of hydrogel swelling behavior on drug release was assessed. The swelling ratios for Dual_Dox_Fibre25@cGel and Dual_Dox_Fibre100@cGel were found to increase with time initially and plateau after day 1 (Table S4), which were consistent with previous reports on similar types of Pluronic and cellulose composite hydrogel systems.^41^ In spite of the hydrogel swelling, we observed only a marginal decrease in Dox released from the Dual_Dox_Fibre@cGels compared to free Dual_Dox_Fibres (Figures 3C and 5C), which could be attributed to the interactions between the released Dox and cGel components, as previously discussed. It is thus evident that the swelling behavior of the hydrogel does not substantially affect Dox release. Instead, the drug release kinetics are primarily governed by the pore system within the fibres, and the composite hydrogel thus serves as a useful depot for the Dox-loaded fibres.

Next, we evaluated the rheological properties of the hydrogel formulations by performing oscillatory sweeps. During a frequency sweep at 1% strain, Fibre100@cGel and cGel showed higher storage modulus (*G*′) than loss modulus (*G*″) values over 0.1–10 Hz, hence confirming their viscoelastic behaviors (Figure S9). The storage modulus of Fibre100@cGel (11,890 Pa) was found to be close to that of pure cGel (12,890 Pa). Additionally, both Fibre100@cGel and cGel maintained their viscoelastic properties up to 5% shear strain (Figure 5D). These results indicate that the inclusion of the fibrous nanoparticle superstructures into the hydrogel matrix did not adversely affect the mechanical properties of the composite hydrogel, indicating good mixing compatibility between the two different components. Thixotropy is a desirable property for injectable hydrogel implants in biomedical applications. The shear-thinning properties of preformed hydrogels facilitate the ease of application by injection using a syringe and needle. Upon removal of shear stress, the reformation of the polymeric networks and the recovery of mechanical properties allow the hydrogels to perform their intended function at the site of administration. At high shear strains (1000%), Fibre100@cGel showed a nearly 2000-fold decrease in mechanical stiffness (*G*′ < *G*″; Figure 5E). Close to 100% recovery of *G*′ was seen when the shear strain was reduced to 1%, hence indicating the reformation of polymeric networks within the hydrogel and the restoration of viscoelastic behavior. This demonstrates that the Fibre100@cGel recovers its mechanical properties and maintains its function after deformation. Overall, these results suggest that the nanoparticle superstructure-loaded cGel formulation holds promise as an injectable and implantable drug delivery system, owing to its shear thinning behavior and ability to recover its mechanical properties while still maintaining the drug release control of the fibres.

### 3.5 Evaluation of In Vitro Biocompatibility and the Sustained Anticancer Effect

**Figure 6:**
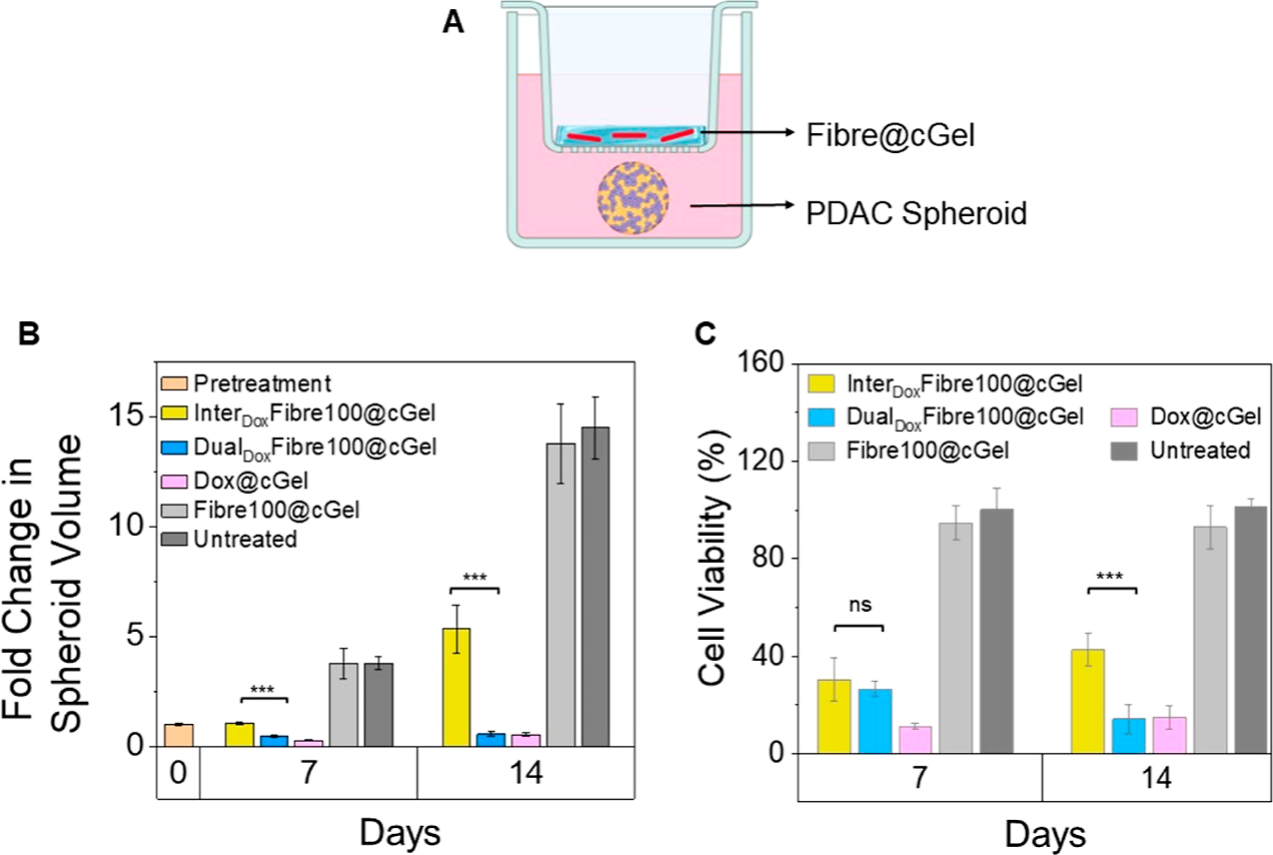
Anticancer efficacy and biocompatibility of Dox-loaded Fibre@cGel formulations in 3D PDAC spheroid models. (A) Schematic representation of the experimental setup. (B) Fold change in spheroid volume on days 7 and 14 post-treatment compared to day 0. (C) Cell viability relative to untreated controls determined using the WST-1 cell viability assay at days 7 and 14 post-treatment. Data points represent mean ± SD of two independent experiments (*n* = 3). ***P < 0.001.

Next, we investigated the potential of the Dual_Dox_Fibre@cGel formulation as a long-lasting drug-eluting depot to prevent tumor recurrence. A 3D spheroid model of pancreatic ductal adenocarcinoma (PDAC) derived from coculturing human pancreatic ductal adenocarcinoma PANC-1 cells with fibroblastic PSC, which more accurately recapitulates the dense fibrotic stroma, as well as the complex cell-extracellular matrix and cell–cell interactions observed in vivo, was employed.^42^ As illustrated in Figure 6A, hydrogels containing the Dox-loaded 3D nanoparticle assemblies were added as a uniform layer to the upper compartment of the transwell inserts, which were then placed in the wells containing spheroids. The spheroids were treated with Dual_Dox_Fibre100@cGel and Inter_Dox_Fibre100@cGel for up to 14 days. Empty Fibre100@cGel without Dox loading was included as a control to demonstrate the biocompatibility of the experiment.

The Dual_Dox_Fibre100@cGel formulation showed a strong and sustained anticancer effect, with a significant reduction in spheroid volume by approximately 0.5- and 0.4-fold on days 7 and 14 of treatment compared to day 0 (Figures 6B and S10). Treatment of the spheroids with Inter_Dox_Fibre100@cGel over 7 days did not lead to a decrease in spheroid volume but instead induced growth suppression, resulting only in a marginal 1.1-fold increase in spheroid volume compared to day 0. By day 14, the Inter_Dox_Fibre100@cGel-treated spheroids had regrown considerably, with a 5.3-fold increase in volume. The disparity in the anticancer efficacy between Dual_Dox_Fibre100@cGel and Inter_Dox_Fibre100@cGel can be attributed to differences in the drug release profiles of the two formulations. As seen from Figure 3D,F, both formulations released Dox for 7 days, resulting in significant initial suppression of spheroid growth. A greater spheroid growth inhibition was seen with Dual_Dox_Fibre100@cGel due to its higher Dox release than Inter_Dox_Fibre100@cGel at day 7. Beyond this time point, Dual_Dox_Fibre100@cGel continued to release Dox, while Inter_Dox_Fibre100@cGel showed minimal or no further Dox release. This led to the sustained antitumor effect and reduction in the initial spheroid volume seen with Dual_Dox_Fibre100@cGel, whereas tumor cell regrowth and an increase in spheroid volume were observed for spheroids treated with Inter_Dox_Fibre100@cGel by day 14. In the case of Dox@cGel, a greater reduction in spheroid volume by 0.7-fold at day 7 was observed compared with Dual_Dox_Fibre100@cGel and Inter_Dox_Fibre100@cGel (Figure 6B). As seen from Figure 5C, the lack of a drug release control mechanism in the Dox@cGel formulation resulted in a rapid burst release, with 70% of Dox released within 24 h, reaching a plateau at approximately 80% on day 5. The rapid and excessive release of Dox thus led to a strong anticancer effect on the spheroids in vitro by day 7. Despite the anticancer effect seen, the large and uncontrolled burst release of drugs from implants is undesirable as it may lead to overdose, truncated, and erratic drug release profiles, which could lead to unwanted toxicity in healthy tissues in vivo.^43^ In contrast, the prolonged release of lower amounts of Dox from the Dual_Dox_Fibre100@cGel could potentially offer benefits for achieving a sustained anticancer effect while inducing lower toxicities in vivo, as seen from previous studies.^44,45^ To evaluate biocompatibility, Fibre100@cGel, which is composed of empty Fibre100 dispersed within the composite hydrogel, was included as a control. The Fibre100@cGel-treated spheroids did not exhibit significant growth inhibition compared to the untreated control (*P* > 0.05). As seen from Figure 6B, the volumes of the Fibre100@cGel-treated spheroids increased by 3.7- and 13.8-fold compared to 3.7- and 14.7-fold for the untreated control on days 7 and 14, respectively. These results thus indicate that the components of the Fibre@cGel formulation do not induce significant toxicity in the absence of Dox and are biocompatible.

An end-point cell viability assessment of spheroids was also performed on days 7 and 14 post-treatment. Spheroids treated with Dual_Dox_Fibre100@cGel showed significant cell death with a cell viability of 26.5 ± 2.7% relative to the untreated control on day 7 (Figure 6C). The cytotoxicity was further increased by day 14, with a cell viability of 14.2 ± 4.9%, clearly demonstrating the sustained anticancer effects due to continued Dox release over an extended period. On the other hand, Inter_Dox_Fibre100@cGel gave significant cell death on day 7, with a cell viability of 30.4 ± 7.6%. However, on day 14, the cell viability increased slightly to 39.6 ± 8.0%. This decrease in cytotoxicity by day 14 could be due to the cessation of Dox release after 7 days, which limited the sustained anticancer effect. Consistent with the effect on spheroid volume, Dox@cGel induced elevated cell death at day 7, with a cell viability of 11.3 ± 1.3%, due to the large burst release of Dox from the hydrogel. With minimal drug released from the Dox@cGel after day 7, a marginal rise in cell viability to 14.8 ± 4.8% was observed on day 14, which is comparable to that attained by Dual_Dox_Fibre100@cGel. Importantly, Fibre100@cGel showed no toxicity at both time points, further confirming the biocompatibility of the formulation. Taken together, these findings provide compelling evidence that the sustained drug release profile of the Dual_Dox_Fibre100@cGel formulation exhibits superior therapeutic efficacy compared to Inter_Dox_Fibre100@cGel and gives rise to a similar anticancer effect as Dox@cGel.

**Figure 7:**
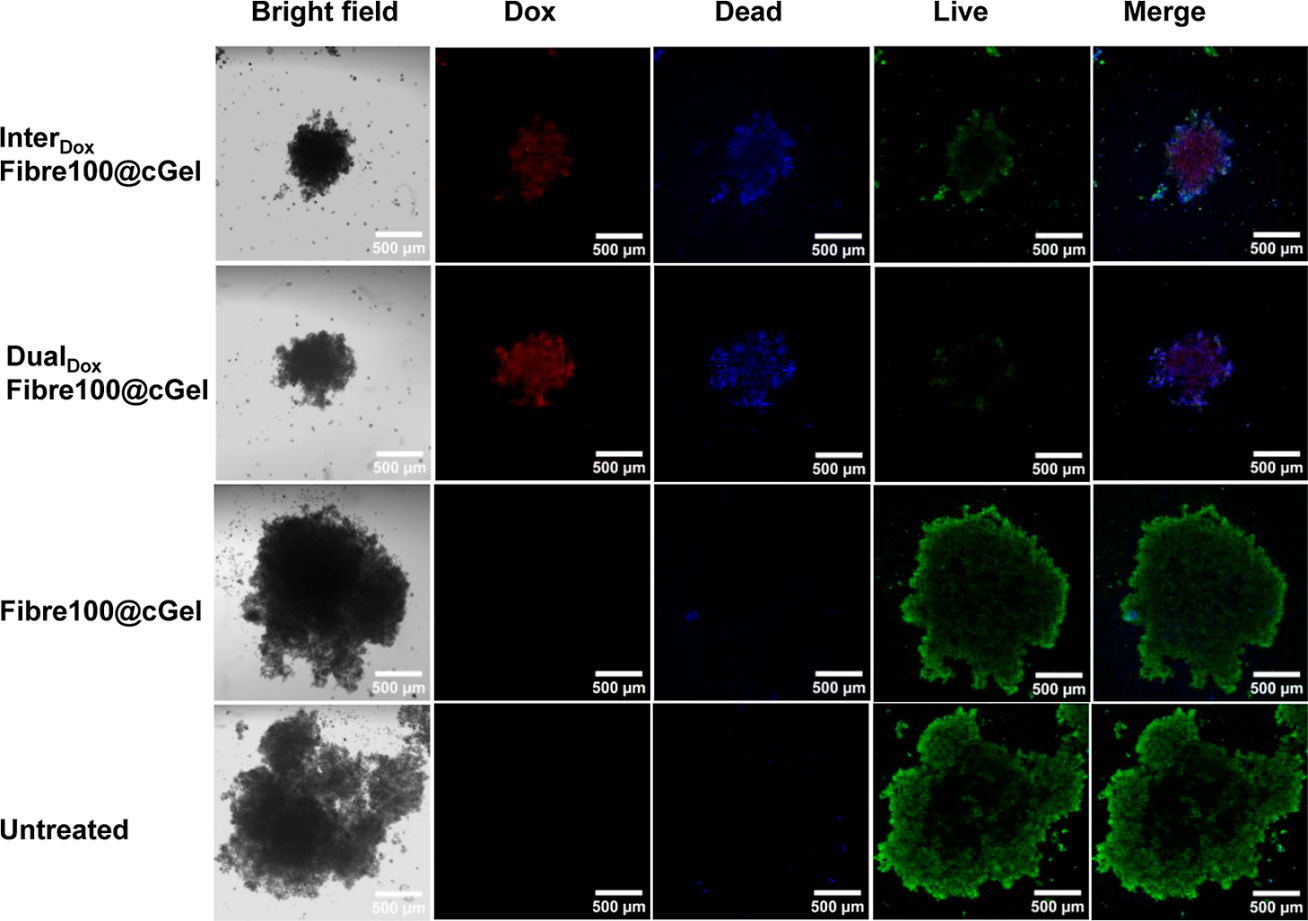
Confocal images of PDAC spheroids treated for 7 days with Inter_Dox_Fibre100@cGel, Dual_Dox_Fibre100@cGel, and Fibre100@cGel. Green and blue represent viable and nonviable cells, respectively.

Fluorescence-based live–dead cell viability staining was performed to establish a relationship between the volume changes observed with the spheroids and the cell viability following treatment. On days 7 and 14 post-treatment, the spheroids were stained with the live/dead dyes, which enable clear differentiation between viable (green fluorescence) and nonviable (blue fluorescence) cells through confocal microscopy (Figures 7 and S10). First, the red fluorescence observed in the Dual_Dox_Fibre100@cGel and Inter_Dox_Fibre100@cGel-treated spheroids indicates the successful release of Dox from the hydrogel formulations, followed by uptake and distribution within the spheroids. Comparatively, the Dual_Dox_Fibre100@cGel treatment led to slightly higher red fluorescence signals within the spheroid, consistent with the enhanced Dox release from this formulation compared to Inter_Dox_Fibre100@cGel. Subsequent cell death was evidenced by blue fluorescence signals in both Dual_Dox_Fibre100@cGel and Inter_Dox_Fibre100@cGel-treated spheroids, whereas spheroids treated with Fibre100@cGel and untreated controls showed no or minimal signals at days 7 and 4. Spheroids treated with Dual_Dox_Fibre100@cGel showed reduced green fluorescence compared to those treated with Inter_Dox_Fibre100@cGel, hence indicating the superior therapeutic effect of the former due to enhanced Dox release. These results corresponded well with the pronounced decrease in spheroid volume observed for the Dual_Dox_Fibre100@cGel formulation compared to Inter_Dox_Fibre100@cGel (Figure 6B). Furthermore, the Fibre100@cGel-treated spheroids showed similar levels of green fluorescence compared to the untreated controls, thus demonstrating formulation biocompatibility. Overall, these nanoparticle superstructure hydrogel formulations hold great potential as implants for preventing postsurgical tumor recurrence, which is a major cause of treatment failure in many solid tumors.^46,47^

## 4 Conclusions

In this study, we have introduced a novel approach for the fabrication of hierarchically porous 3D nanoparticle superstructures using a directional freezing-driven colloidal assembly. By controlling the freezing rate, size, and concentration of the PSiNP building blocks, we were able to produce self-assembled 3D fibrous structures from the PSiNPs, and the resulting interparticle pores could be easily tuned by selecting appropriate nanoparticle sizes without the need for any organic solvents, emulsifying agents, or chemical modifications. For the first time, we have demonstrated the potential of these fibrous materials produced by directional freezing for controlled drug release applications by entrapping drug molecules of different sizes (Dox and Lyz) in the larger interparticle meso/macropores and smaller PSiNP micropores within the fibres. The hierarchical porosity of the fibres allowed for sustained drug release for up to 25 days, with faster release from the larger interparticle meso/macropores followed by slower drug release from the smaller PSiNP micropores. Additionally, we have shown that these fibres can be formulated into hydrogels, which could be used as injectable/implantable drug delivery systems. Overall, our approach provides a versatile platform to produce hierarchical porous scaffolds with a tunable porosity and large surface areas. These unique features of the 3D nanoparticle assemblies do offer opportunities for various applications beyond cancer drug delivery, such as the temporal release of therapeutics to promote wound healing and the enhancement of enzymatic or chemical catalysis.

## Supporting Information Available

Freezing time, Dox loading efficiency, physicochemical characterization data for PSiNP building blocks, FE-SEM images of fibres, PXRD diffraction patterns, rheology data, and morphological changes to the PDAC spheroid following treatment (bright-field and confocal images) (PDF)
