# Supplementary material for: Free-Standing Hierarchically Porous Silica Nanoparticle Superstructures: Bridging the Nano- to Microscale for Tailorable Delivery of Small and Large Therapeutics
Source: ACS Appl Mater Interfaces. 2024 Jan 25;16(5):5568–81. doi: 10.1021/acsami.3c16463 (PMC10859928; doi:10.1021/acsami.3c16463)
Supplement: Supplementary file 1 — am3c16463_si_001.pdf [file am3c16463_si_001.pdf]

Supporting Information

Free-Standing Hierarchically Porous Silica Nanoparticle Superstructures: Bridging the Nano- to Microscale for Tailorable Delivery of Small and Large Therapeutics

Sandeep Palvai,[†] *Delanyo Kpeglo,*[†] *George Newham,*[†] *Sally A. Peyman,*[†‡] *Stephen D. Evans,*[†] *and Zhan Yuin Ong*[*†‡]

† School of Physics and Astronomy, University of Leeds, Leeds LS2 9JT, U.K.

‡ Leeds Institute of Biomedical and Clinical Sciences, School of Medicine, University of Leeds, Leeds LS2 9JT, U.K.

* E-mail: Z.Y.Ong@leeds.ac.uk

Synthesis and Characterisation of Porous Silica Nanoparticles (PSiNPs). Highly uniform PSiNPs of approximately 25, 50, 100, and 150 nm diameters were synthesised using a one-pot polyelectrolyte complex templated method.[1,2] Briefly, arginine/poly(acrylic acid) (Arg/PAA) polyelectrolyte complexes were formed by adding 2.5 mL of 1.1 x 10^{-1} mM PAA and 7.5 mL of 8.6 x 10^{-2} mM Arg to 40 mL of ultrapure water. The solution was dispersed in an ultrasonic bath for 5 min before the final volume was topped up to 250 mL with IPA. The mixture was left to stir for 1 h at room temperature (22 °C). Subsequently, 1, 2, 6 and 9 mL of TEOS was added and the mixture was stirred overnight at room temperature to obtain PSiNPs of approximately 25, 50, 100 and 150 nm in diameters, respectively. The PSiNPs were collected by centrifugation (17,000 × g for 1 h) and purified by rinsing four times with ultrapure water. FITC labelled 100 nm PSiNPs were prepared by adding approximately 0.1 mol % of FITC-PEG-silane and 6.5 mL of TEOS to a final volume of 250 mL of PEC templates in 80 % v/v IPA.

The hydrodynamic diameters and zeta-potentials PSiNPs were measured using dynamic light scattering (DLS) on a Zetasizer Nano instrument equipped with a He-Ne laser and scattered light detection at an angle of 173° (Malvern Instruments Ltd., Worcestershire, UK). Each sample was analysed three times, with 12 measurements per run conducted at 25 °C. The mean size and zeta-potential values reported as the average of the three runs, along with the corresponding standard deviation. The PSiNP morphology was analysed using high-resolution TEM on an FEI Tecnai G2-Spirit instrument operating at an accelerating voltage of 120 kV, with image acquisition performed using a Gatan Ultrascan 4000 CCD camera. 5 μL of each dispersion was pipetted onto a carbon-coated copper grid and allowed to air-dry at room temperature prior to imaging. The size distribution of the PSiNPs was determined from the TEM images using ImageJ.

References

[1] G. Newham, R. K. Mathew, H. Wurdak, S. D. Evans, Z. Y. Ong, *J. Colloid Interface Sci.* **2021**, *584*, 669

[2] G. Newham, S. D. Evans, Z. Y. Ong, *J. Colloid Interface Sci.* **2022**, *617*, 224.

Table S1. Freezing time observed for PSiNP dispersions of various diameters and concentrations at –22 and -196 °C.

Freezing temperature	PSiNP concentration (wt. %)	Freezing time for PSiNP dispersions (min:sec)			
		25 nm	50 nm	100 nm	150 nm
-22 °C	0.25	14:36 ± 00:37	14:11± 00:37	16:43 ± 00:45	16:18 ± 00:37
	0.5	15:06 ± 00:28	14:49 ± 01:01	16:56 ± 01:04	17:02 ± 00:59
	1.0	16:22 ± 00:34	18:39 ± 02:52	18:03 ± 01:16	17:31 ± 00:50
-196 °C	0.5	01:45 ± 00:04	1:51 ± 00:16	1:51 ± 00:08	1:58 ± 00:10

Table S2. Loading Efficiency of Dox into the interparticle pores of the fibres by directional freezing of Dox with PSiNPs of various sizes

Fibre type	Loading Efficiency (%)
$Inter_{Dox}Fibre25$	97.72 ± 0.10
$Inter_{Dox}Fibre50$	97.26 ± 0.14
$Inter_{Dox}Fibre100$	96.81 ± 0.62
$Inter_{Dox}Fibre150$	96.40 ± 0.35

Table S3. Loading content of Dox into the micropores of the PSiNPs of various sizes.

PSiNPs Size (nm)	Loading Content (µg mg^{-1})
25	84.27 ± 0.36
50	85.95 ± 0.20
100	89.02 ± 0.96
150	90.51 ± 0.95

Table S4. Swelling ratio of the hydrogel formulations.

Time	$Dual_{Dox}$Fibre25@cGel	$Dual_{Dox}$Fibre100@cGel
6 h	1.3 ± 0.08	1.0 ± 0.1
8 h	1.7 ± 0.3	1.5 ± 0.4
Day 1	2.6 ± 0.4	2.3 ± 0.2
Day 3	2.3 ± 0.1	2.3 ± 0.4
Day 5	1.8 ± 0.3	2.1 ± 0.08
Day 7	1.7 ± 0.3	1.8 ± 0.1

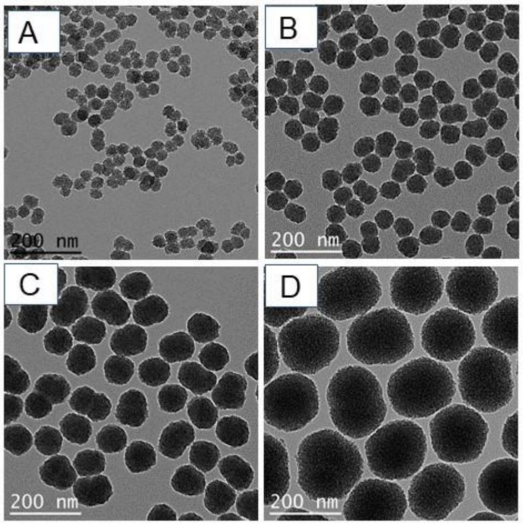

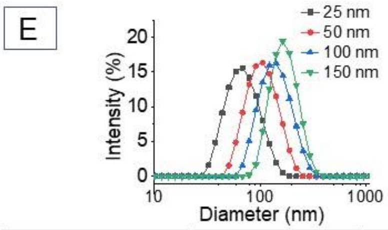

Diameter by TEM ± S.D. (nm) (n=100)	PDI ± S.D.	Zeta Potential ± S.D. (mv)
25.9 ± 3.0	0.106 ± 0.01	- 28.9 ± 5.6
50.9 ± 3.5	0.104 ± 0.01	- 29.9 ± 6.2
100.5 ± 7.5	0.06 ± 0.001	- 32.5 ± 2.8
150.3 ± 8.4	0.11 ± 0.05	- 31.9 ± 5.8

Figure S1. (A-D) TEM images and (E) intensity weighted DLS size distributions for the synthesised PSiNPs. The average diameters determined from the TEM images were (A) 25.9 ± 3.0, (B) 50.9 ± 3.5, (C) 100.5 ± 7.5, and (D) 150.3 ± 8.4 nm (n = 200).

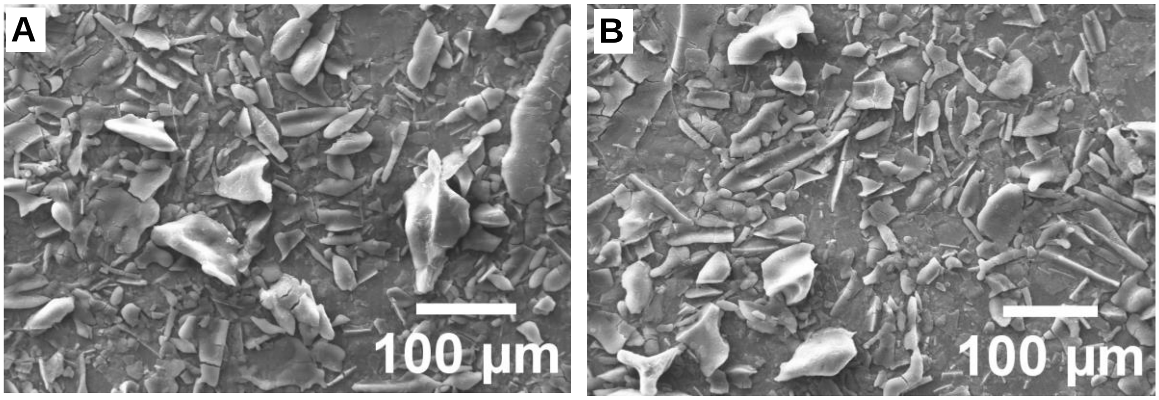

Figure S2. FE-SEM images of structures assembled from 0.5 % w/v of (A) 25 nm (B) 100 nm PSiNPs at -196 °C freezing.

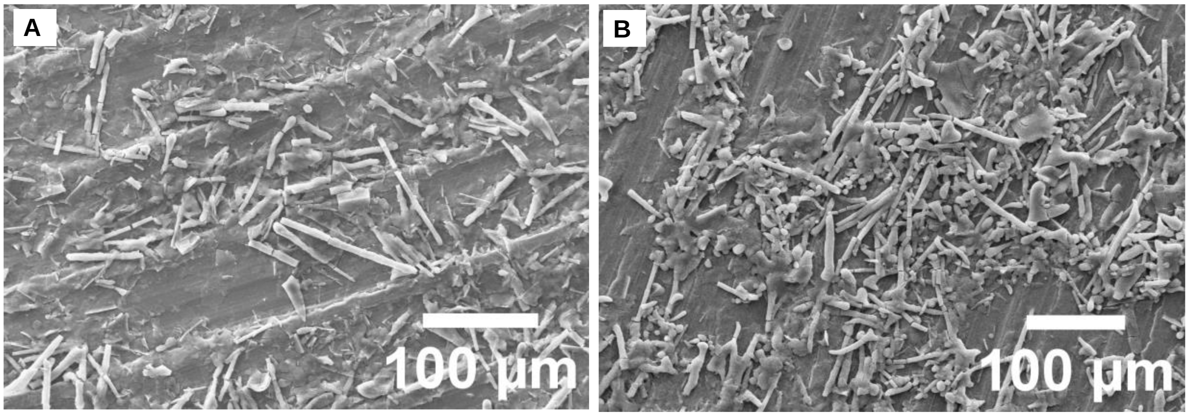

Figure S3. FE-SEM images of fibres assembled from (A) 0.25 % w/v and (B) 1 % w/v of 100 nm PSiNPs.

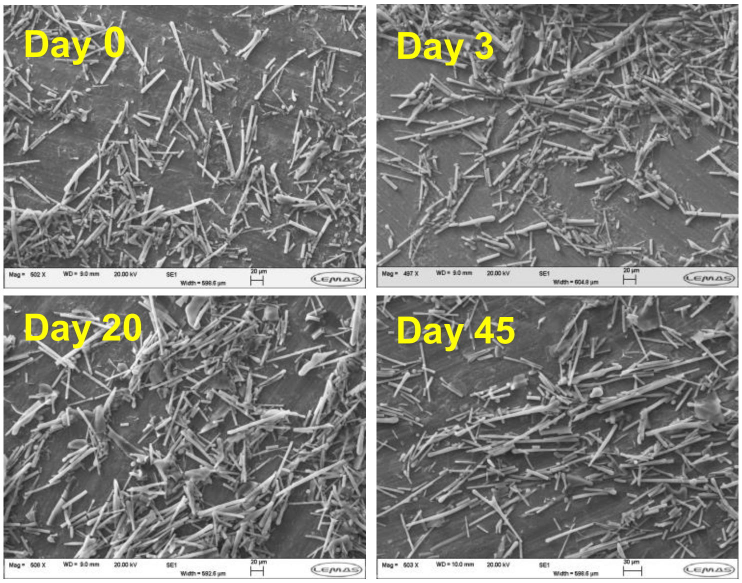

Figure S4. FE-SEM images demonstrating *in vitro* stability of the fibres. Fibres100 (10 mg) were immersed in PBS (2 mL) and subjected to continuous shaking at 200 rpm before being imaged at the indicated time points. The average diameter of the fibres at day 45 was calculated to be 4.6 ± 0.89 μm (n = 60).

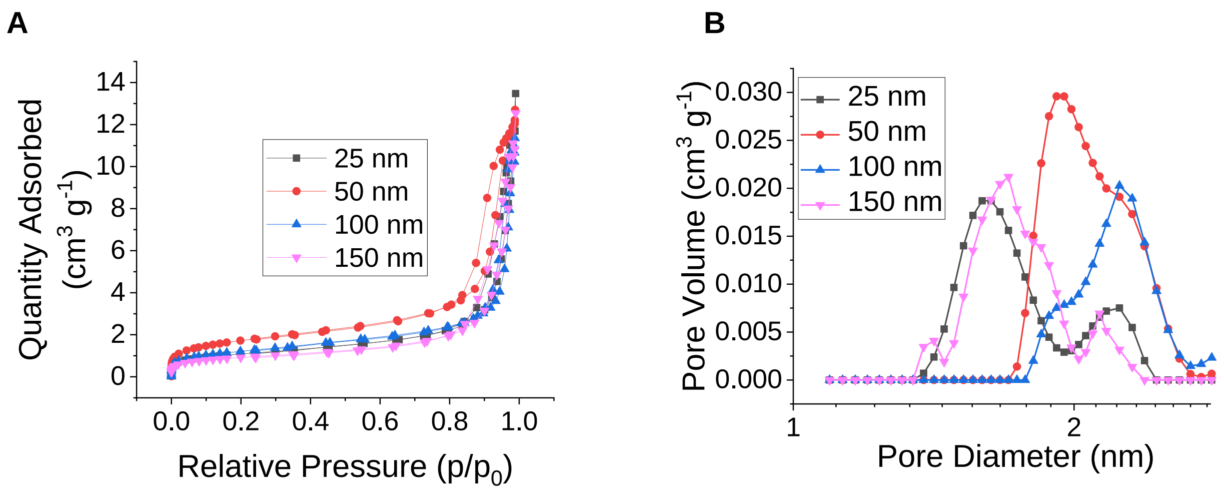

Figure S5. Pore size distribution analysis for 25, 50, 100, and 150 nm PSiNPs by NLDFT analysis.

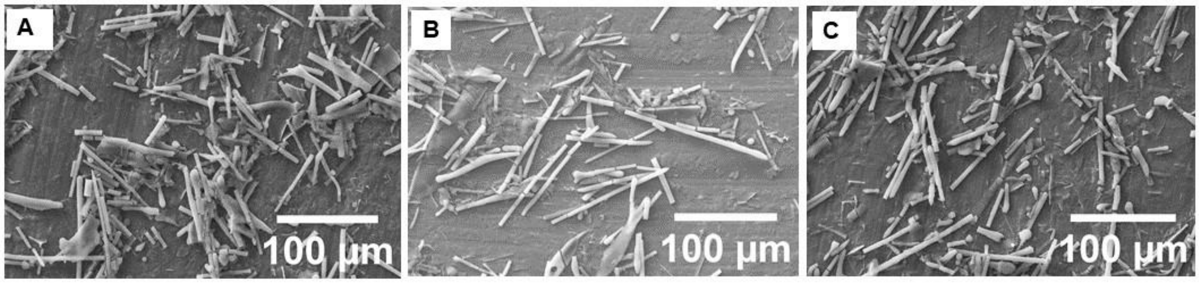

Figure S6. Representative FE-SEM images of Dox loaded fibres. (A) $Inter_{Dox}Fibre100$, (B) $Intra_{Dox}Fibre100$, and (C) $Dual_{Dox}Fibre100$. Fibre diameters were measured to be 5.2 ± 0.7, 4.9 ± 0.7 and 5.2 ± 0.7 for $Inter_{Dox}Fibre100$, $Intra_{Dox}Fibre100$ and $Dual_{Dox}Fibre100$, respectively (n = 60).

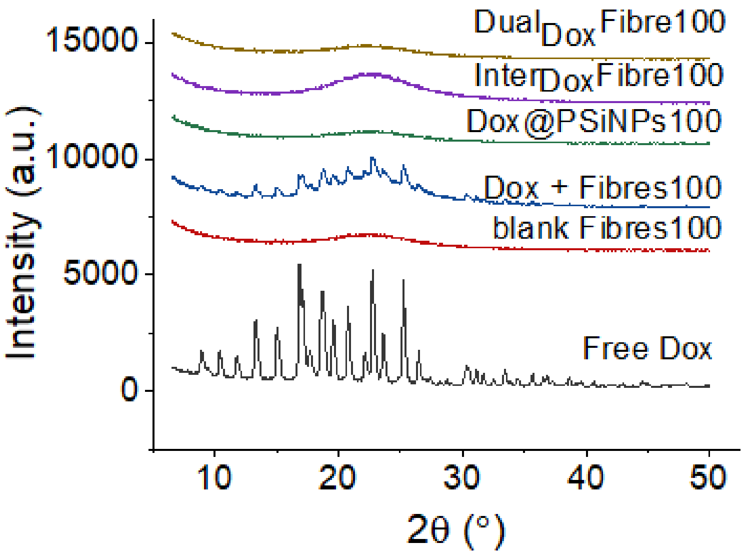

Figure S7. PXRD diffraction patterns for Dox powder and Dox-loaded fibre samples.

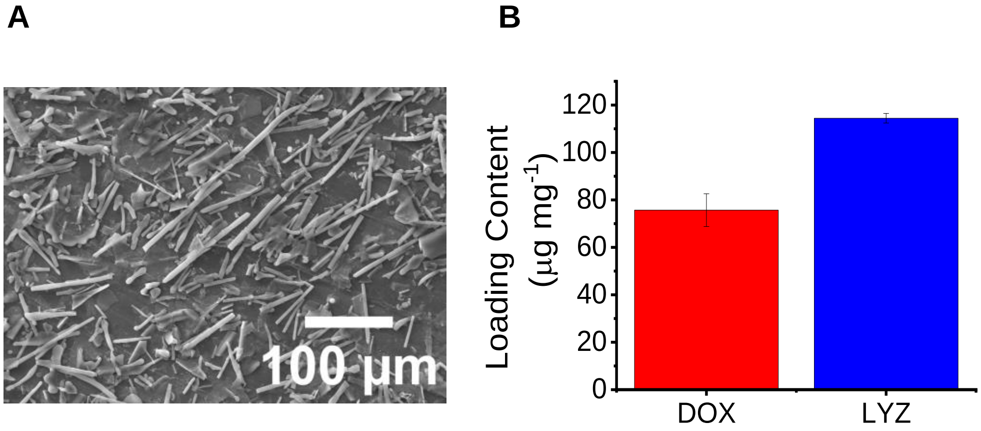

Figure S8. (A) Representative FE-SEM images of Inter$_{Lyz}$Intra$_{Dox}$Fibre100 with co-loading of Lyz in the interparticle mesopores and Dox in the PSiNP micropores and (B) Lyz and Dox loading content within the Inter$_{Lyz}$Intra$_{Dox}$Fibre100. The average diameter of the fibres was calculated to be 5.04 ± 0.92 µm (n = 60).

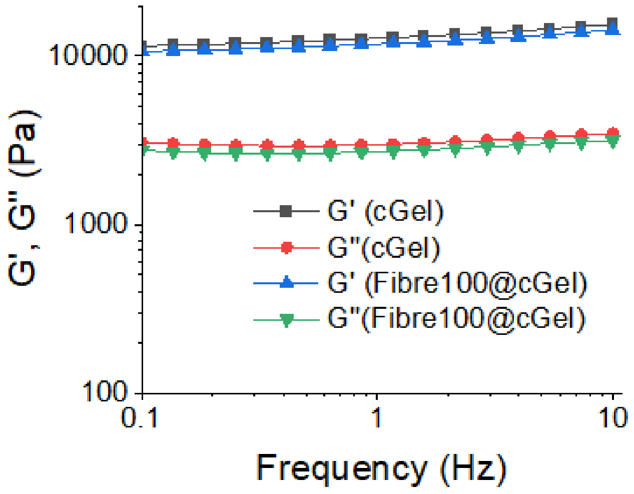

Figure S9. Storage modulus (G') and loss modulus (G") of the Fibre100@cGel and cGel versus frequency sweep (strain 1%).

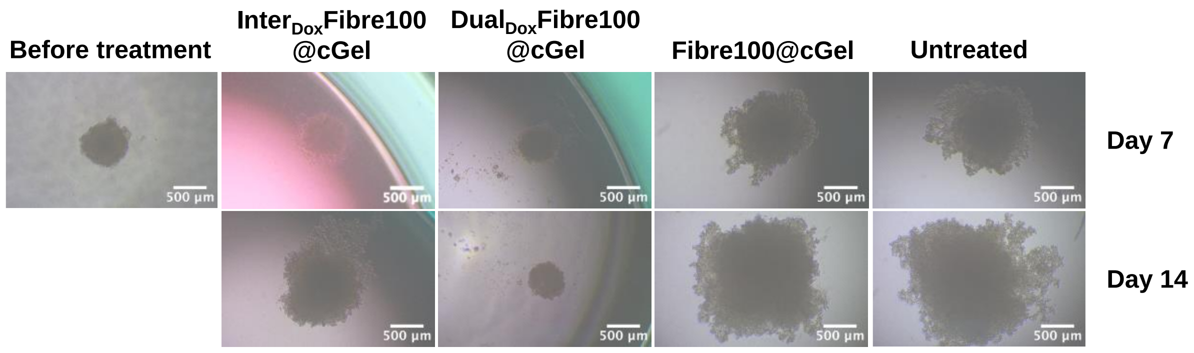

Figure S10. Bright-field images showing the change in spheroid volume with time following treatment with the Dox-loaded Fibre@cGel formulations.

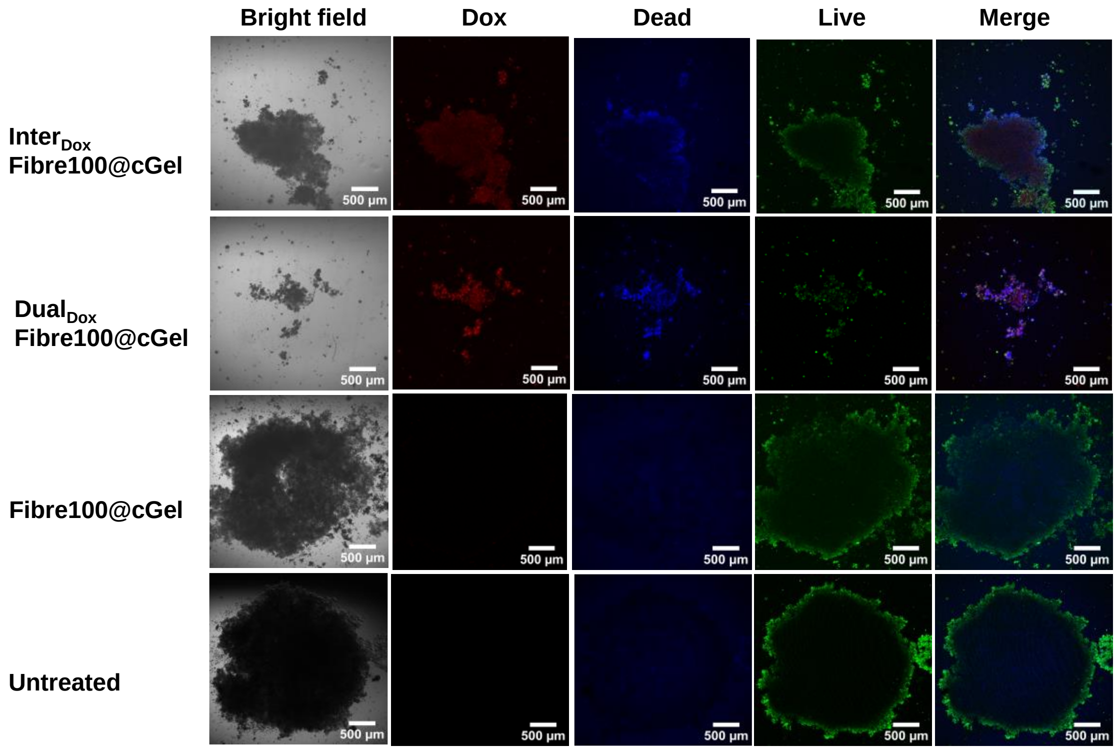

Figure S11. Confocal images of PDAC spheroids treated for 14 days with $Inter_{Dox}$Fibre100@cGel, $Dual_{Dox}$Fibre100@cGel, and Fibre100@cGel. Green and blue, represent viable and non-viable cells, respectively.
